# Supplementary material for: A number-based inventory of size-resolved black carbon particle emissions by global civil aviation
Source: Nat Commun. 2019 Feb 1;10:534. doi: 10.1038/s41467-019-08491-9 (PMC6358618; doi:10.1038/s41467-019-08491-9)
Supplement: Supplementary file 1 — Supplementary Information [file 41467_2019_8491_MOESM1_ESM.pdf]

Supporting information for "A number-based inventory of size-resolved black carbon particle emissions by global civil aviation"

Zhang et al.

Supplementary Figures

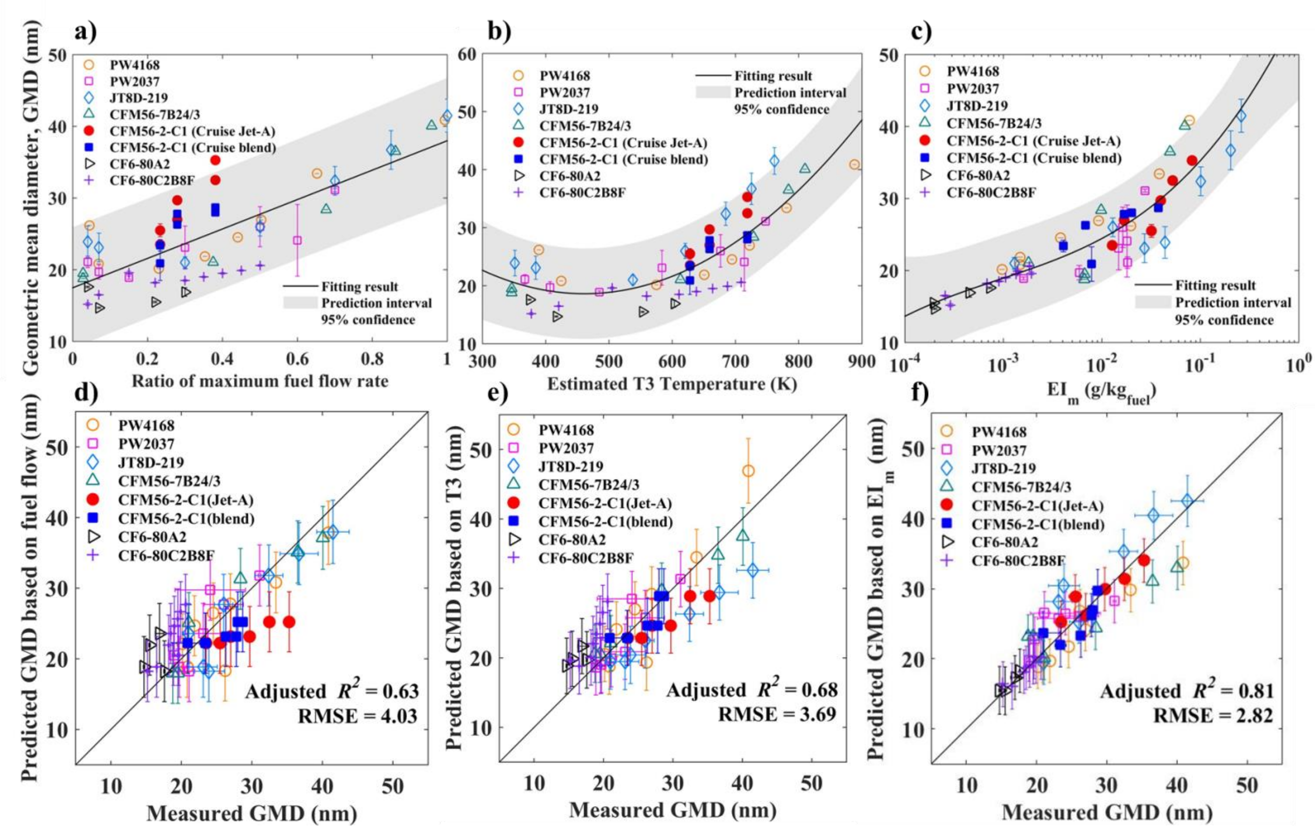

Supplementary Figure 1 Estimation methods for geometric mean diameter (GMD). a) Correlation between GMD and the ratio of maximum fuel flow rate; b) Correlation between GMD and combustor inlet temperature T_3 and c) black carbon mass emission indices EI_m. The shaded area represents the 95% prediction interval. The comparisons among the qualities of the different fitting approaches, namely the goodness of fittings: d) GMD-fuel flow rate; e) GMD-T_3 and f) GMD-EI_m. The vertical error bars represent the standard deviation of the estimations. The horizontal error bars are the uncertainties in the measurements if available.

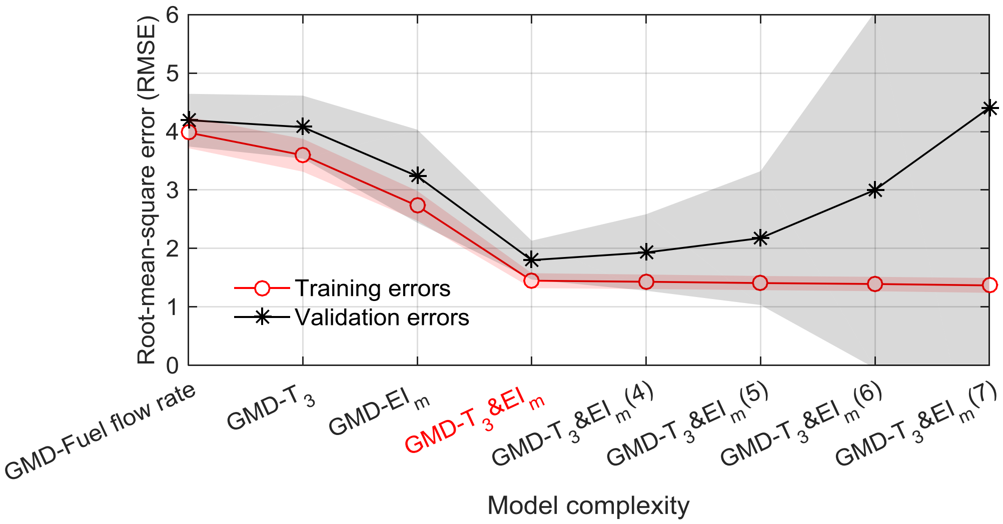

Supplementary Figure 2 Results of the bias-variance trade-off tests.

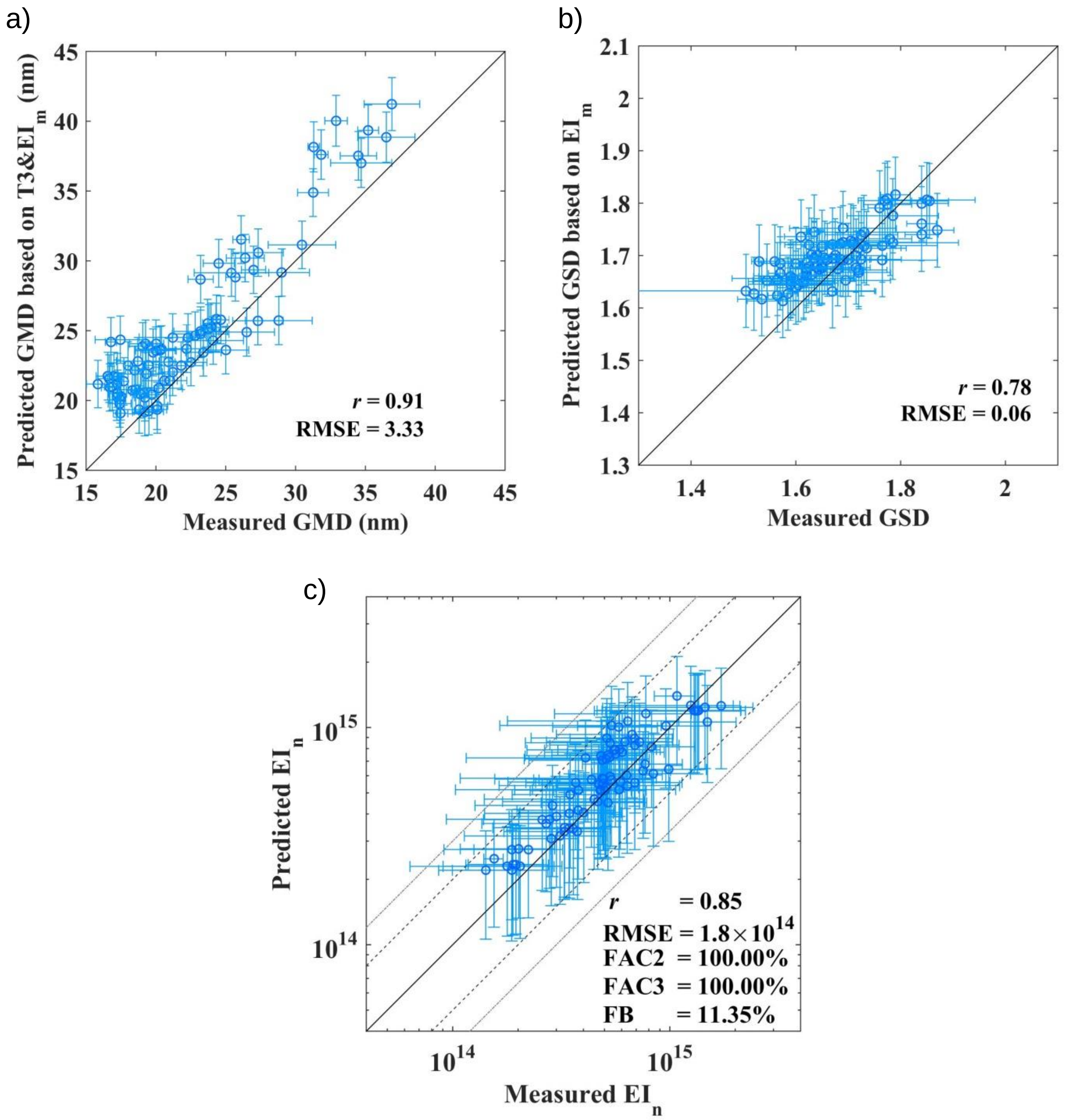

Supplementary Figure 3 Comparison between the model results and APEX data. The measurements are from Aircraft Particle Emissions eXperiment (APEX). a) Comparison between the estimated geometric mean diameters (GMD) and the measurements; b) comparison between the estimated geometric standards (GSD) estimations and the measurements; c) comparison between the black carbon particle number emission indices (EI_n) and the measurements. The vertical error bars represent the standard deviation of the estimations. The horizontal error bars are the uncertainties in the measurements.

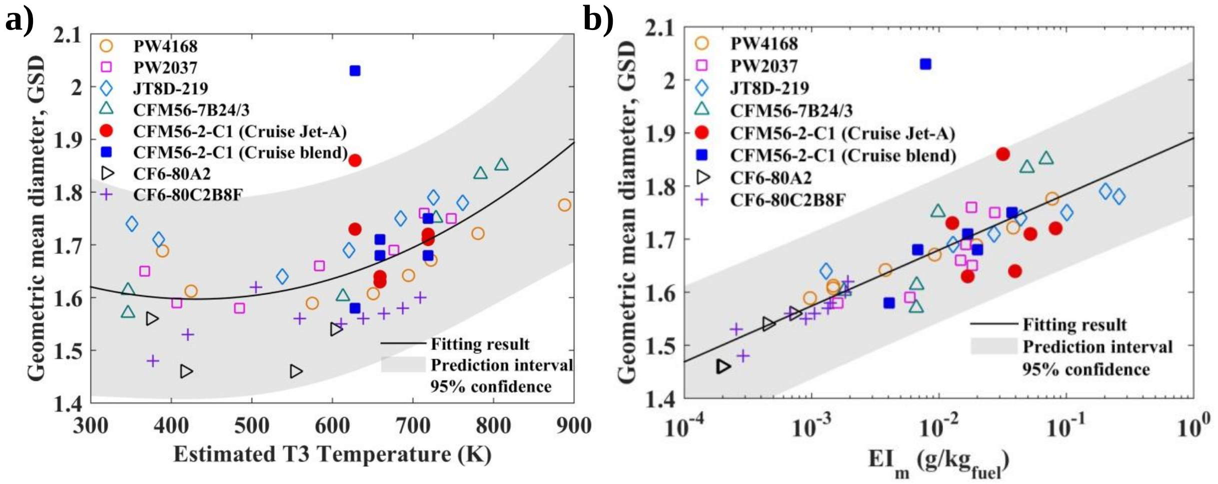

Supplementary Figure 4 Correlations for geometric standard deviation. The correlations between geometric standard deviations (GSD) and the engine parameters: (a) correlation between GSD and combustor inlet temperature T_3; (b) correlation between GSD and black carbon mass emission indices (EI_m). The shaded area is the 95% prediction interval for the fitted correlation.

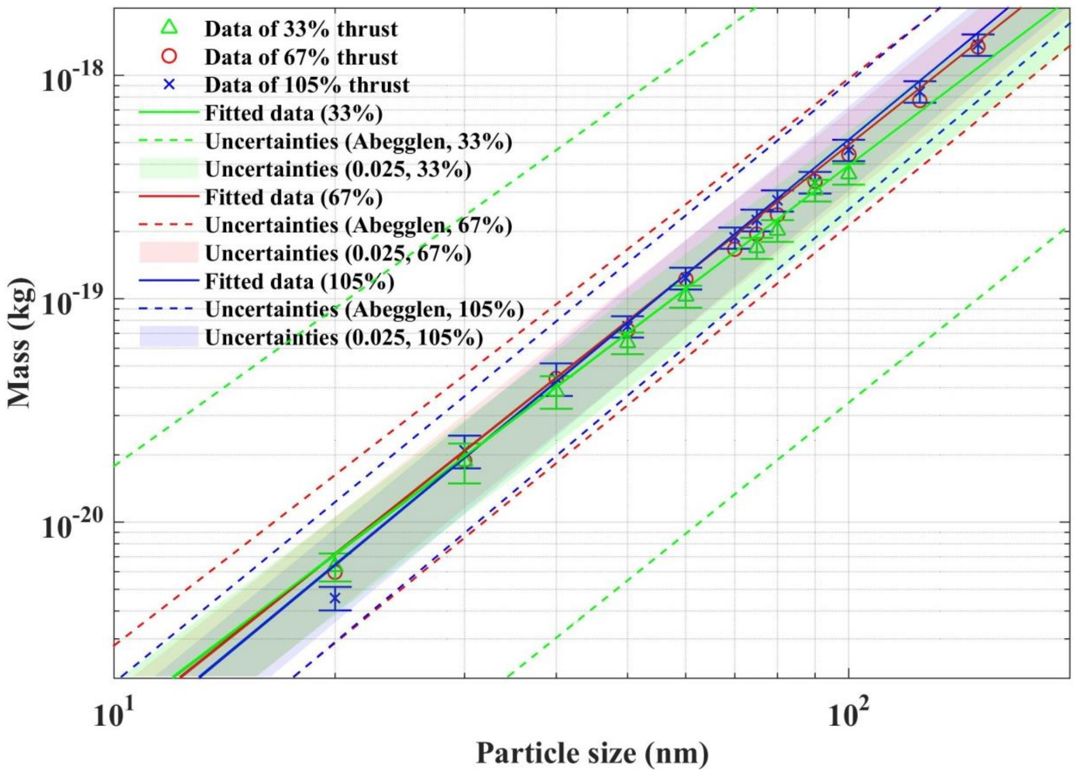

Supplementary Figure 5 Correlation between black carbon particle mass and size. Comparisons between the measured uncertainties of particle mass in Abegglen et al.[39] and the reconstructed uncertainties respectively by the parameters in Abegglen et al.[39] and the adopted uncertainty of ε (0.025). The error bars represent the standard deviation of the measurements.

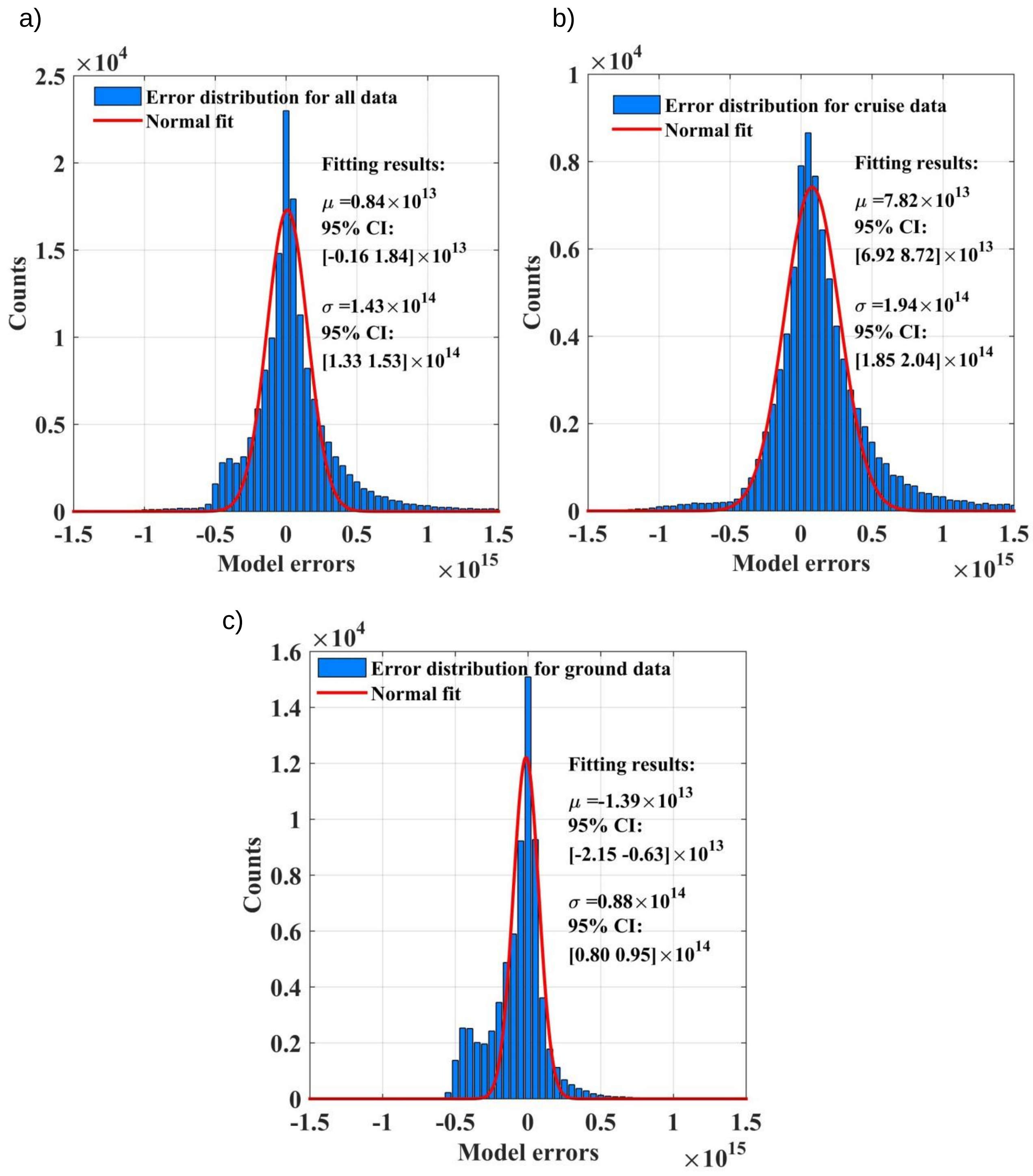

Supplementary Figure 6 Residuals of all the Monte Carlo runs. a) Error distribution for all the data (cruise + ground); b) error distribution for cruise data; c) error distribution for ground data.

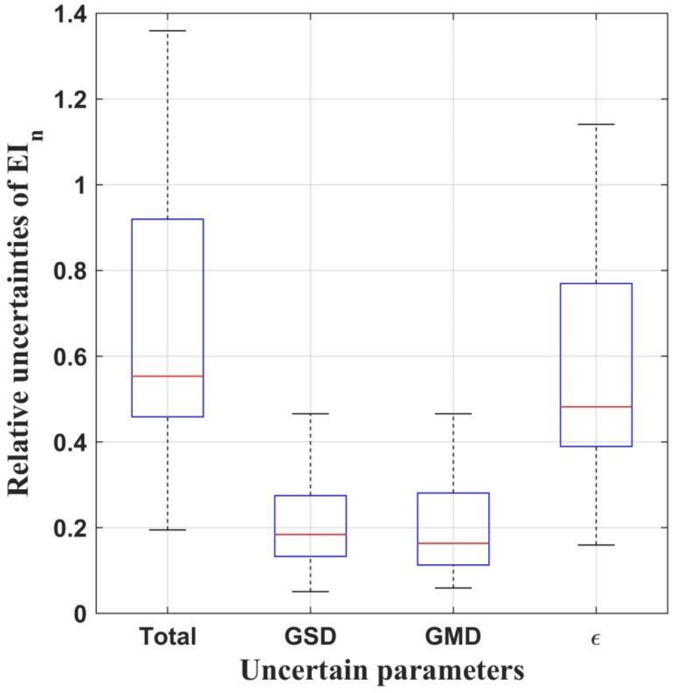

Supplementary Figure 7 Uncertainties caused by the individual uncertain parameters. “Relative” means the predicted value divided by the measured value.

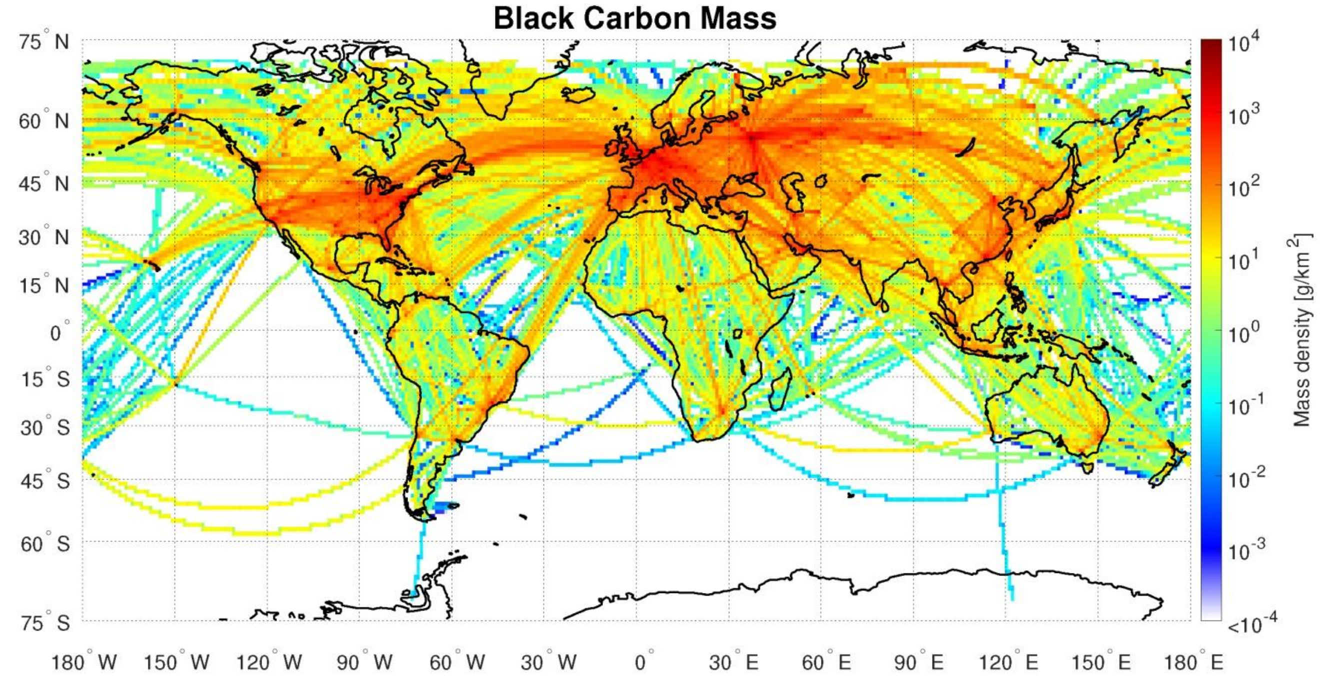

Supplementary Figure 8 Spatial distribution of black carbon mass emission.

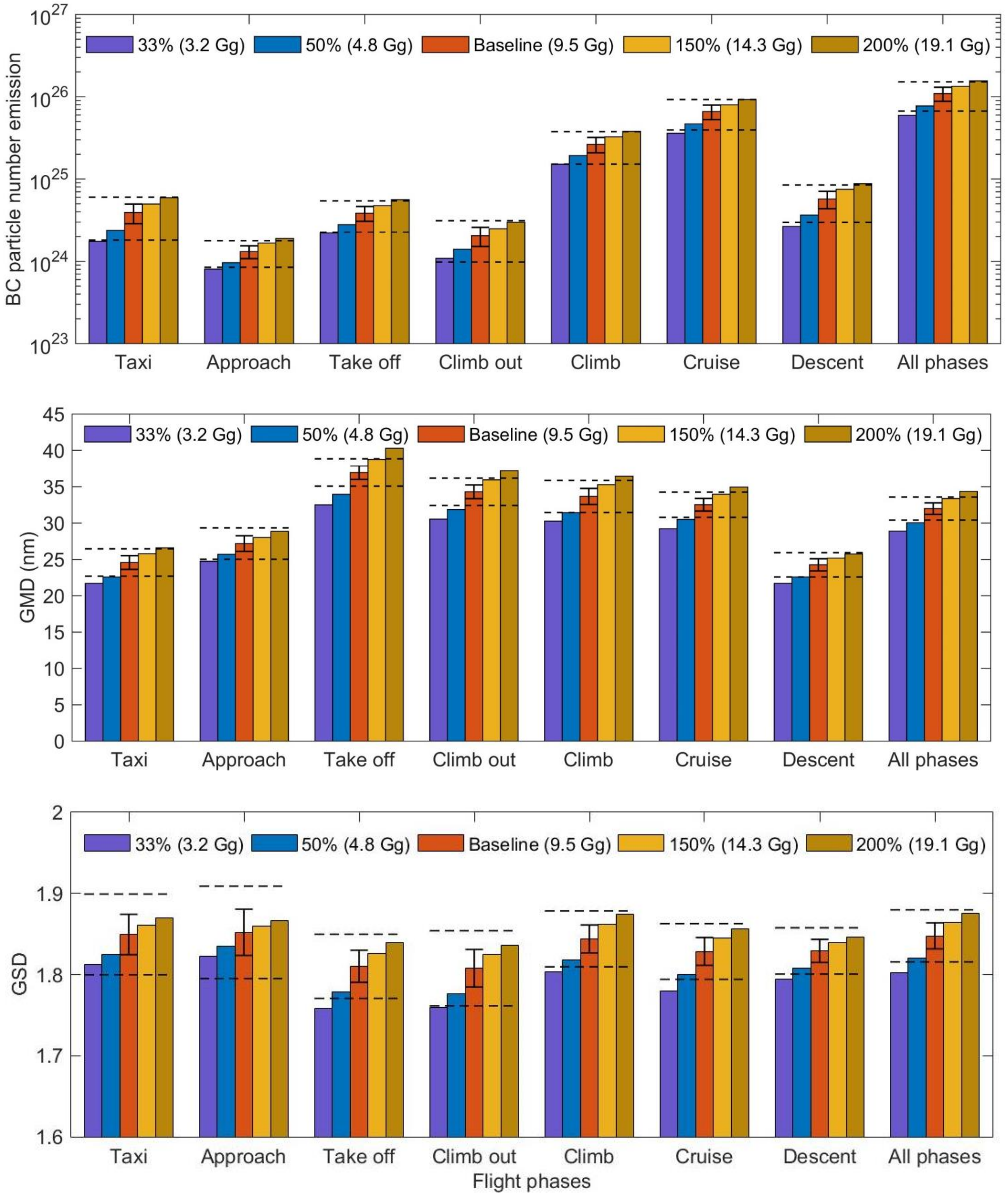

Supplementary Figure 9 Sensitivity tests for black carbon mass emissions. Results of the sensitivity tests for the influences of black carbon (BC) mass emissions. The error bars are the estimated standard deviations of the MC simulated results (68% confidence interval), and the dashed lines are two times of the standard deviation (95% confidence interval).

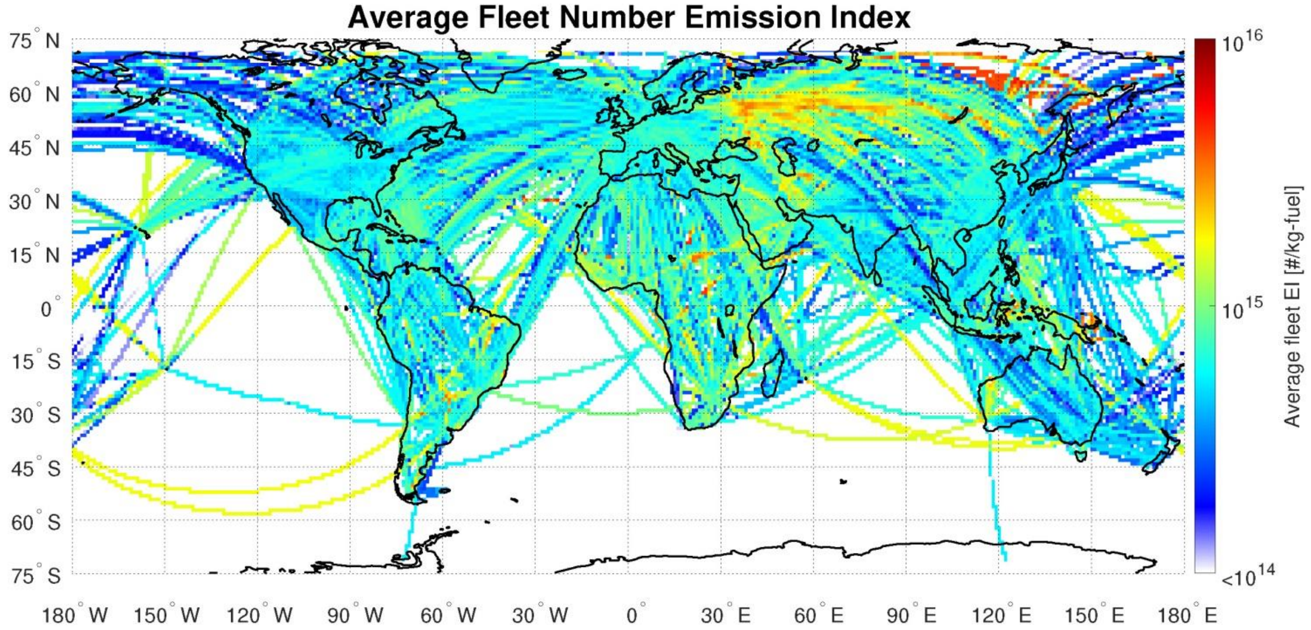

Supplementary Figure 10 Spatial distribution of black carbon number emission indices.

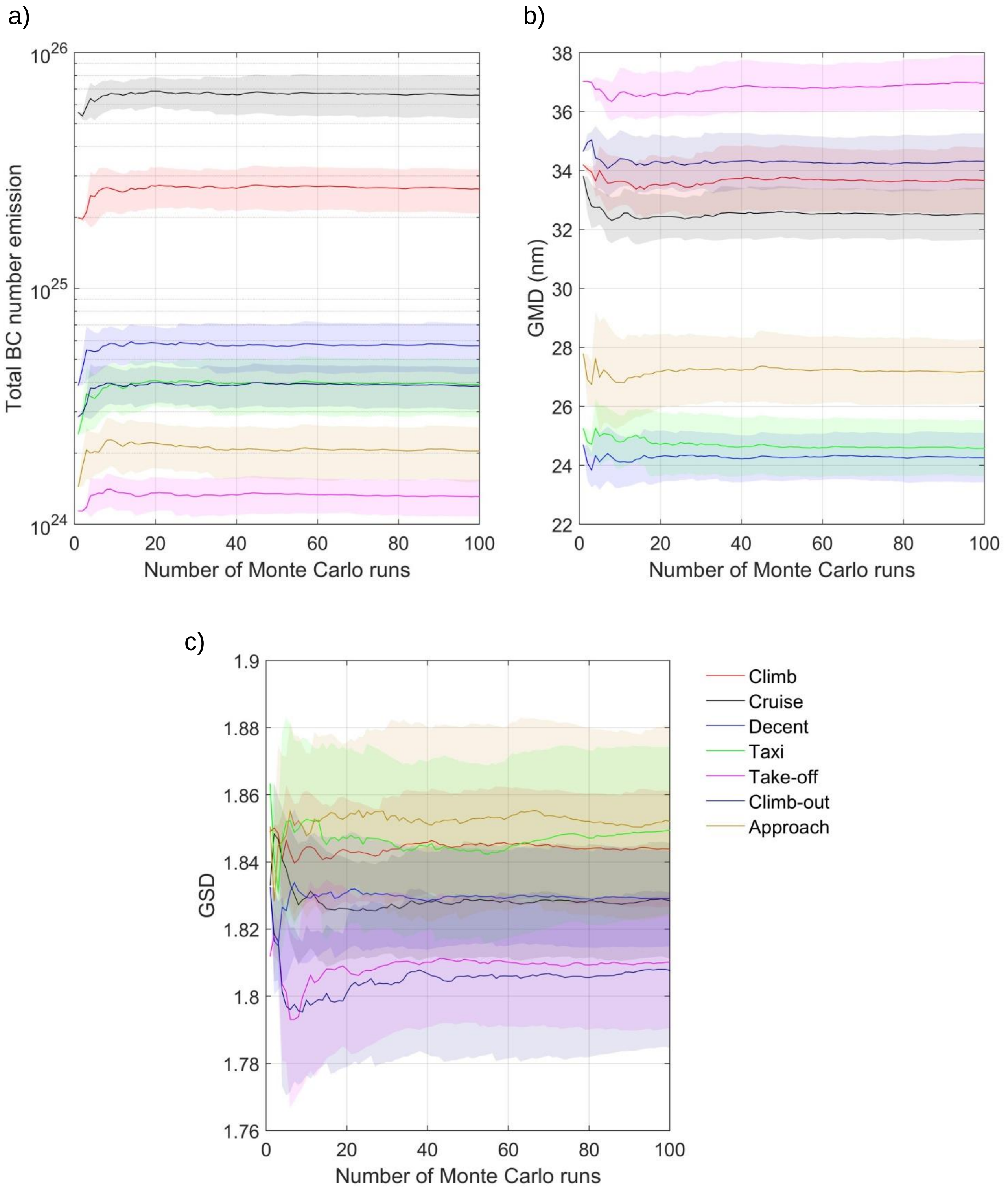

Supplementary Figure 11 Stabilities of the Monte Carlo runs. The results of a) total BC number, b) geometric mean diameter GMD and c) geometric standard deviation GSD with 1 to 100 Monte Carlo runs and the shaded area indicates the standard deviation of all the Monte Carlo runs.

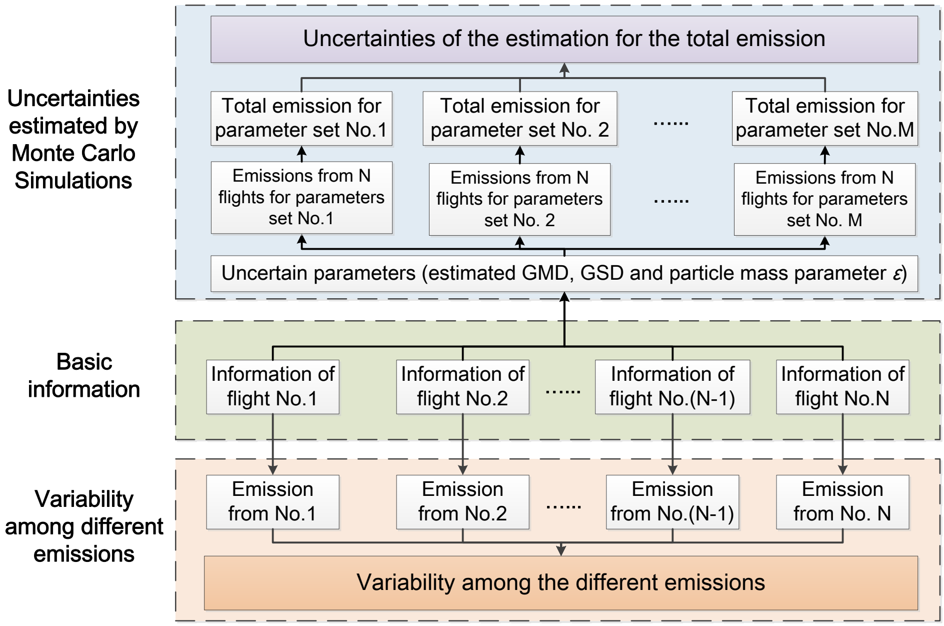

Supplementary Figure 12 Schematic diagram for uncertainty and variability. The differences between the uncertainties of estimations and the variability among different emissions from various engines and flight conditions. Number N represents the amount of the civil flights in one year, about 27.34 million; number M represents of number MC runs, 100 in this study.

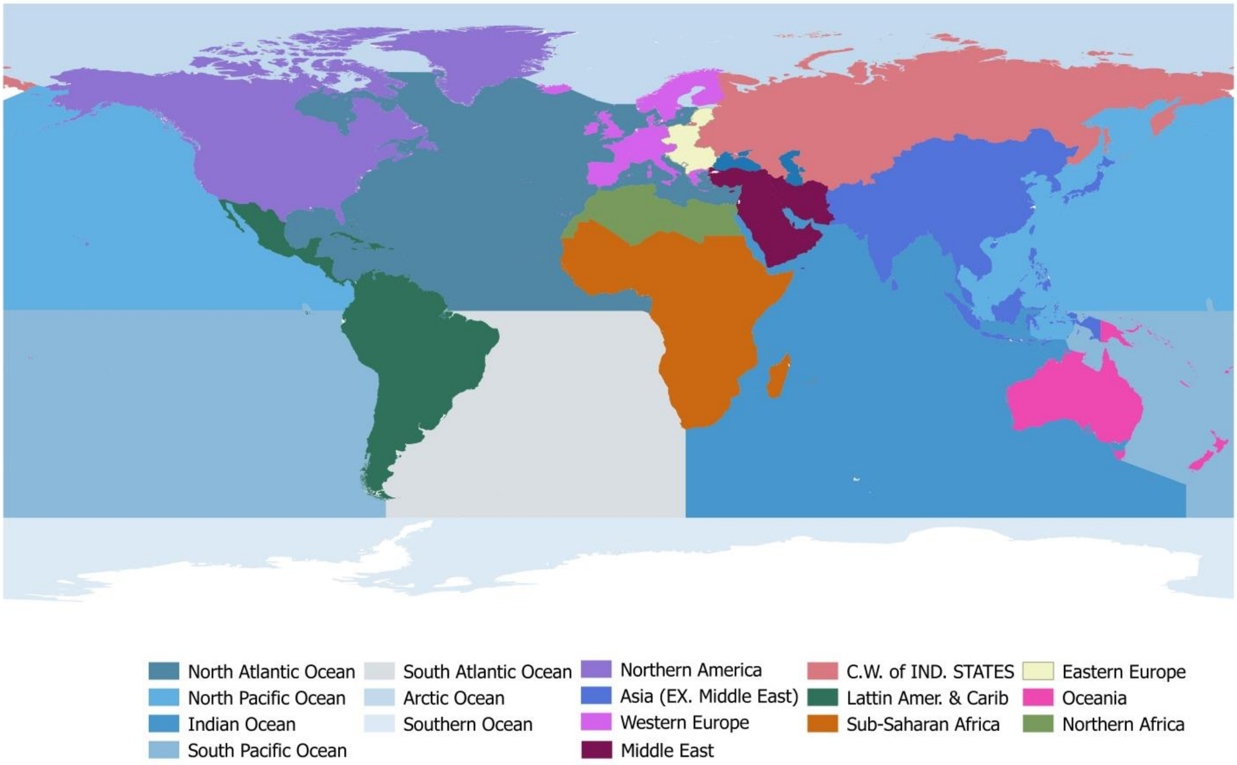

Supplementary Figure 13 Division of different regions

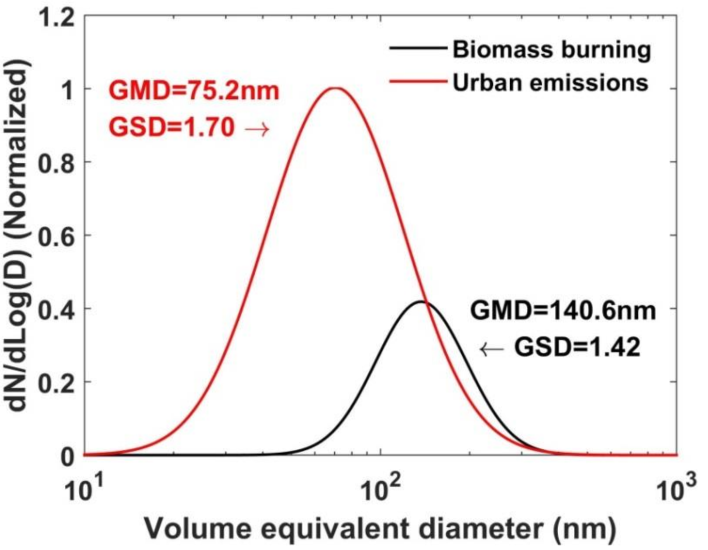

Supplementary Figure 14 Size distributions of the black carbon particles. The particles in urban emissions and open burning [15]

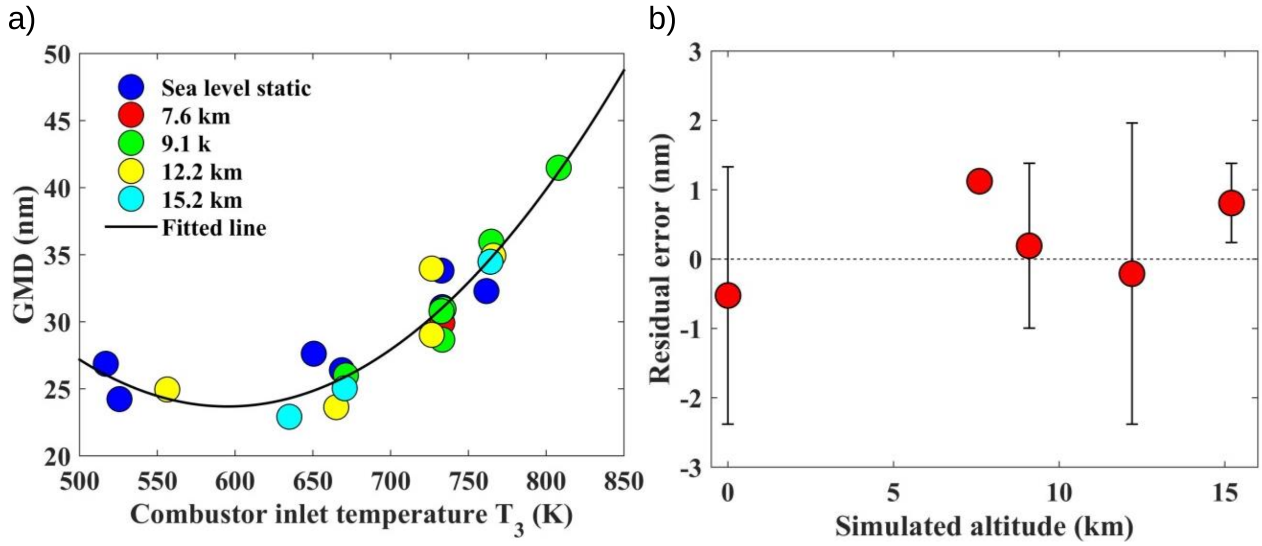

Supplementary Figure 15 Black carbon particle size at different simulated altitudes. The altitudes are from sea level to 15.2 km: (a) Changes of the GMD of BC size distribution with T_3; (b) the residuals between the measurements and the generic fitted relation at different altitudes. The error bars represent the standard deviation of the residual error.

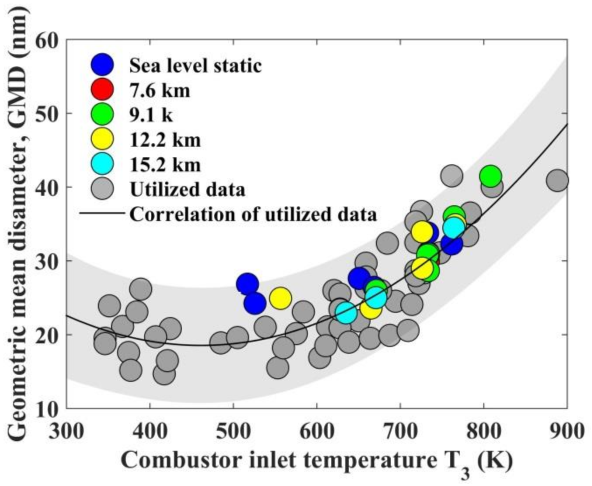

Supplementary Figure 16 Geometric mean diameters at different altitudes. Comparison between the data at different simulated altitudes and the data utilized to develop the correlation between the geometric mean diameter (GMD) and the engine combustor inlet temperature (T_3) (the GMD-T_3 correlation). The shaded area represents the 95% prediction interval.

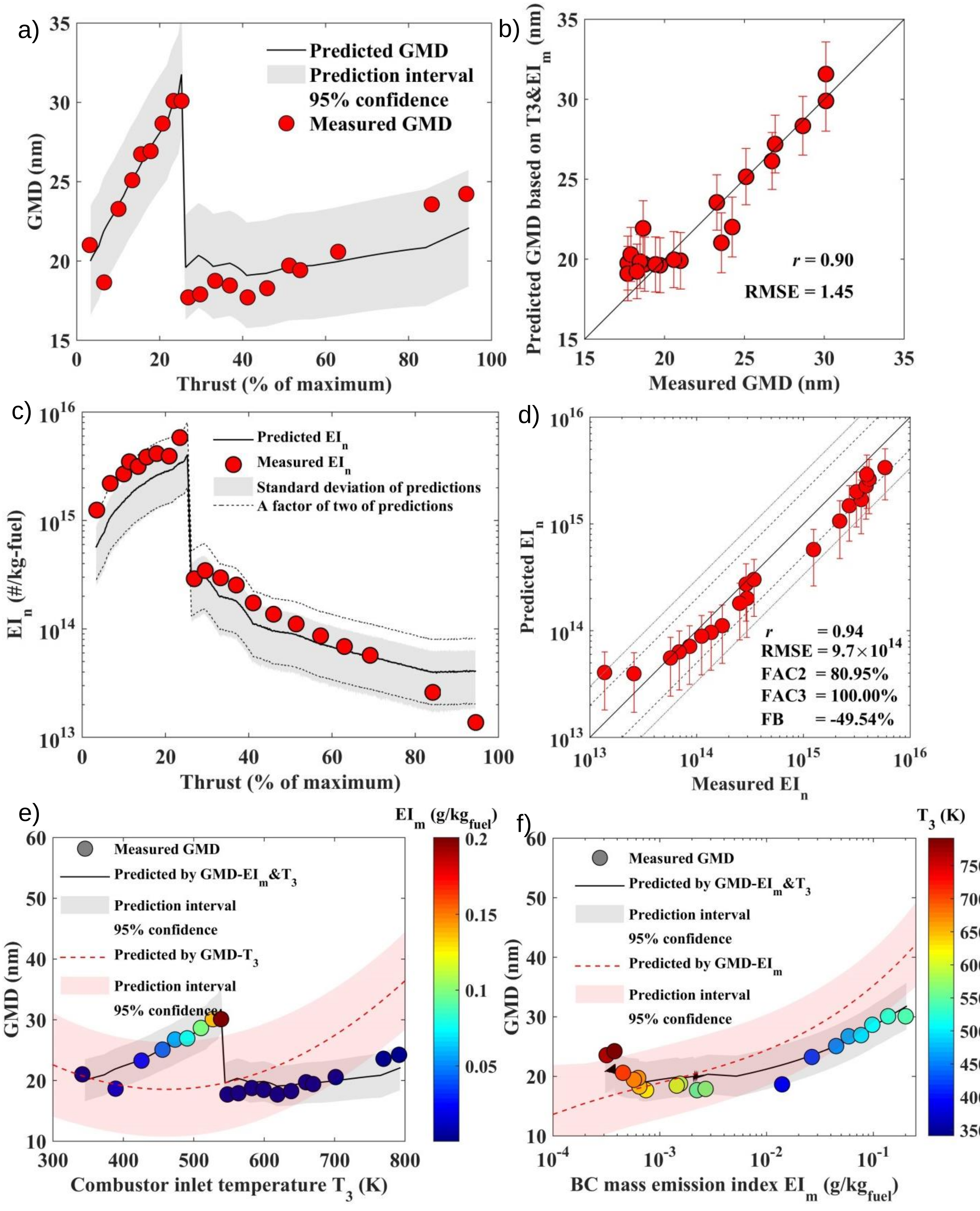

Supplementary Figure 17 Evaluation of the estimations for a double annular combustor. (a) the changes of the GMD with thrust level; (b) the direct comparison between the measured and estimated GMDs; (c) the changes of EI_n(BC) with thrust level; (d) the direct comparison between the measured and estimated EI_n(BC), the dashed lines are 1:2 (or 2:1) ratio lines and the dotted lines are 1:3 (or 3:1) ratio lines; (e) the changes of the GMD with T_3 and the color indicates the BC mass emission indices EI_m; (f) the changes of GMD with EI_m and the color indicates the combustor inlet temperature T_3. Error bars represent the standard deviations of the estimations.

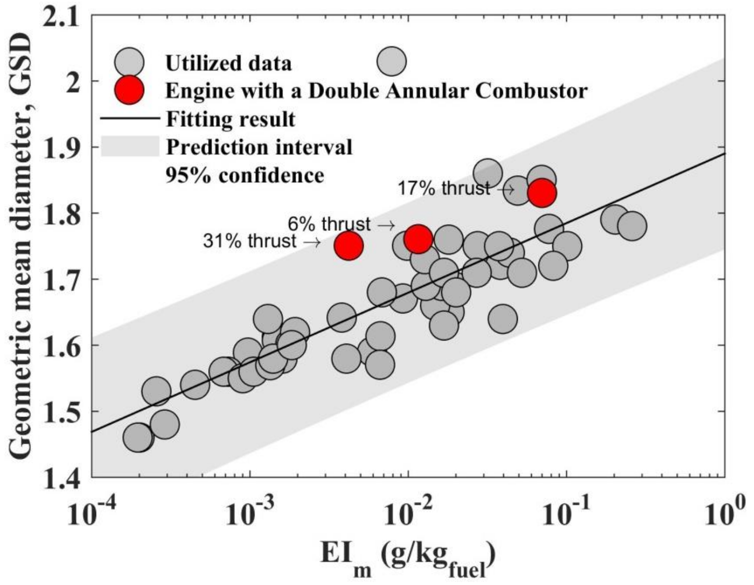

Supplementary Figure 18 Geometric standard deviation (GSD) estimation. Comparison between the measured GSD for the engine with a double annular combustor (CFM56-5B4-2P) and the utilized data to develop the GSD-EI_m(BC) relation. The shaded area represents the 95% prediction interval.

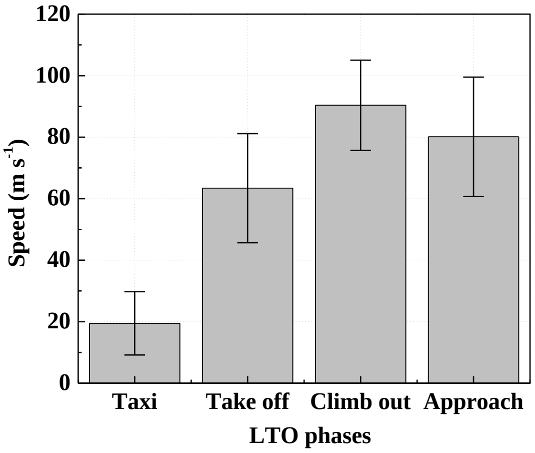

Supplementary Figure 19 Average flight speeds for LTO cycles. The average flight speeds of the sub-phases taxi/take-off/climb-out/approach in the LTO cycle.

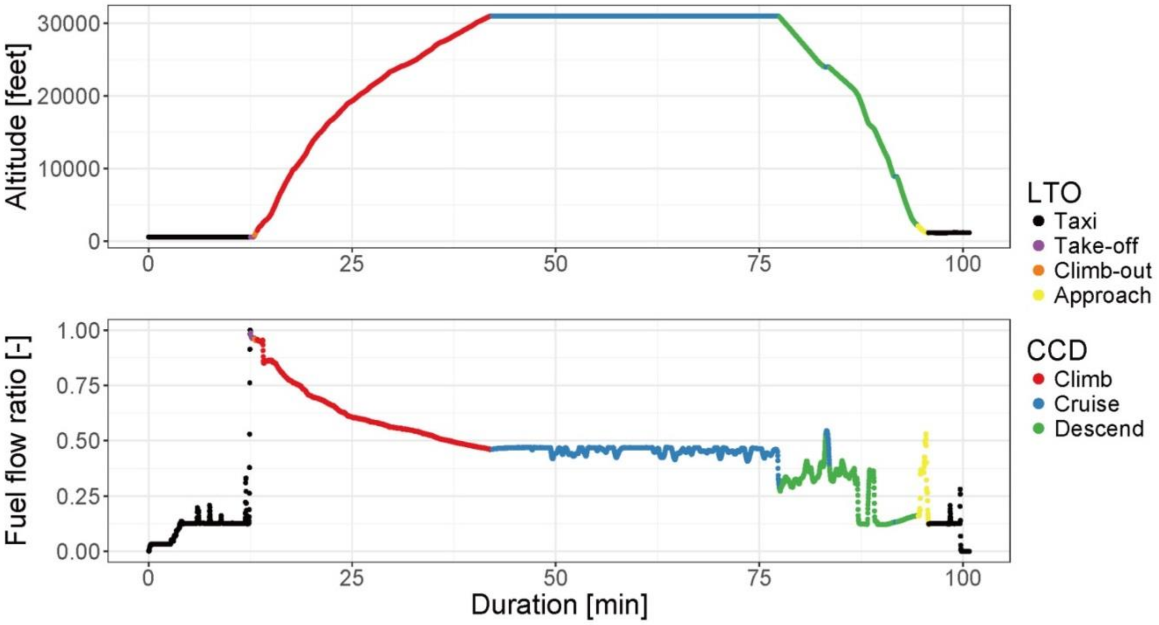

Supplementary Figure 20 An example of the record from Flight Data Recorder. One example of the flight altitude (upper panel) and fuel flow rate normalized by the maximum flow rate at taking off (lower panel).

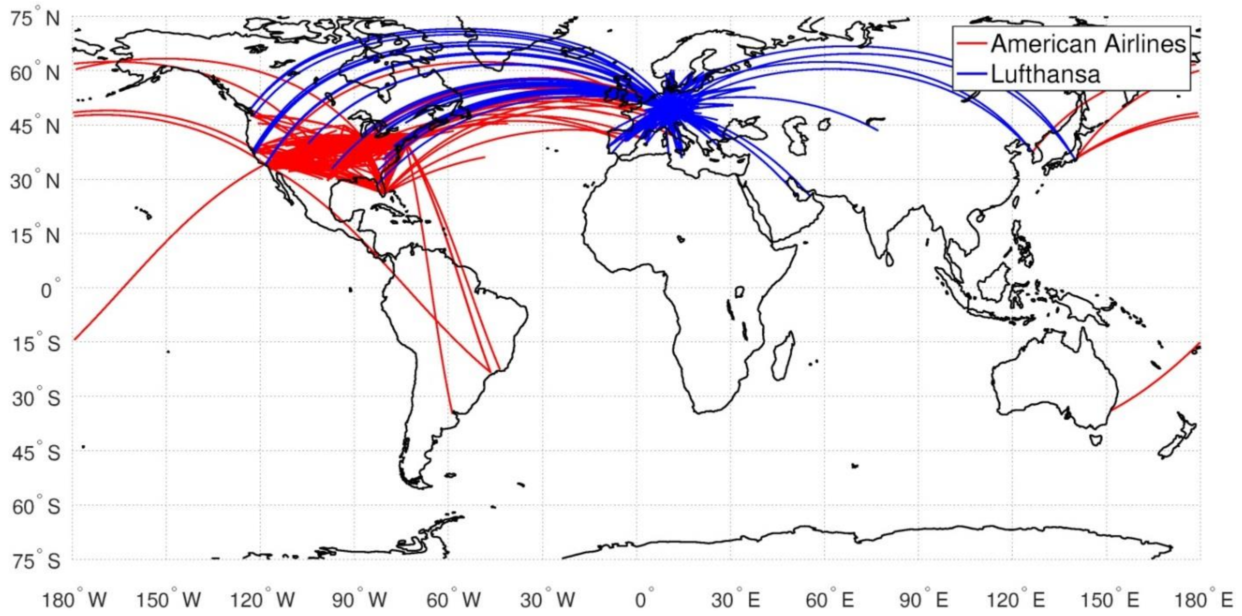

Supplementary Figure 21 Automatic Dependent Surveillance-Broadcast (ADS-B) data. Airlines of the Automatic Dependent Surveillance-Broadcast (ADS-B) data used in this study. The airlines are operated by American Airline (red) and Lufthansa (blue).

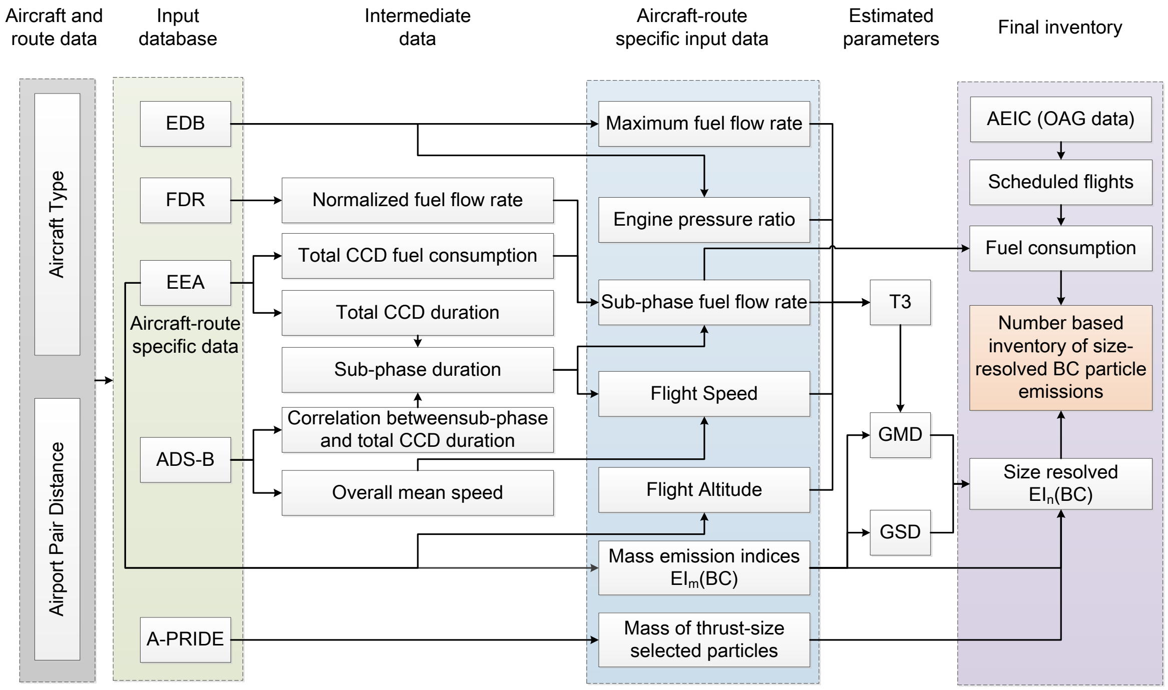

Supplementary Figure 22 Schematic plot for emission inventory development. Simplified flowchart for the development of the number based inventory of size-resolved BC particle emissions.

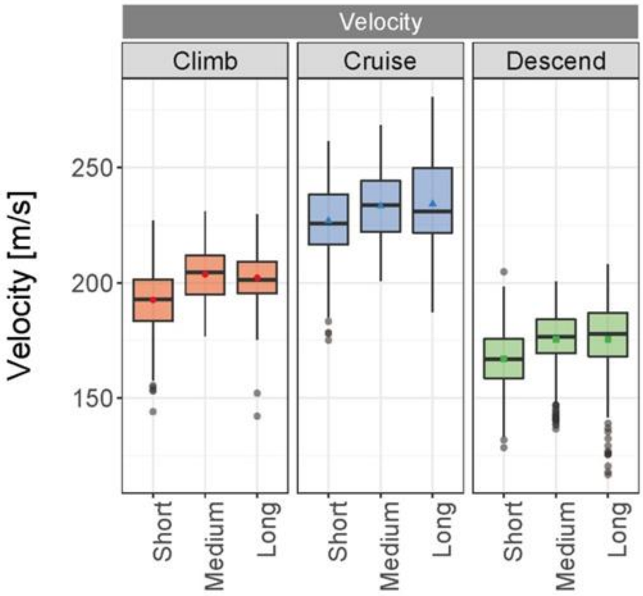

Supplementary Figure 23 Typical flight speeds for CCD phase. Typical flight speed (left panel) and altitude (right panel) for different sub-phases (climb/cruise/descent) in the CCD phase, analyzed based on the ADS-B data.

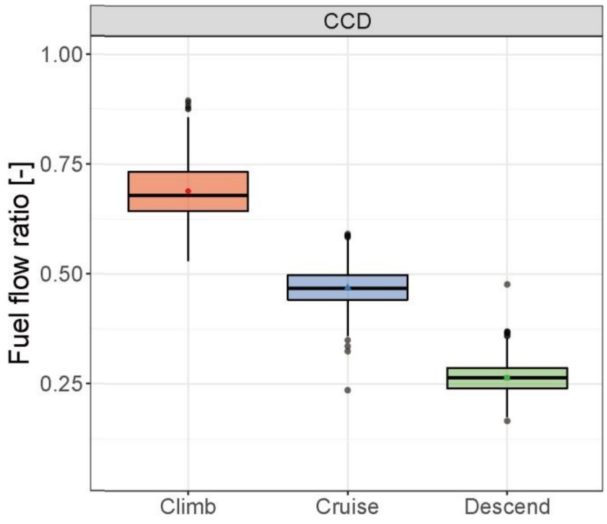

Supplementary Figure 24 Normalized fuel flow rate of different sub-phases.

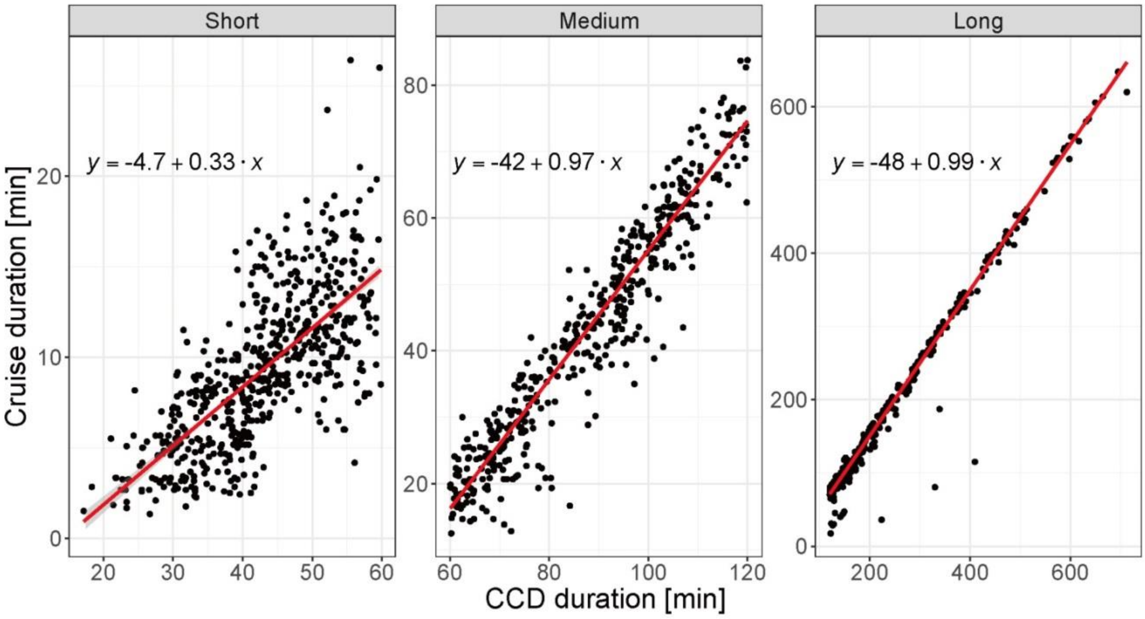

Supplementary Figure 25 Relations between the cruise duration and total CCD duration.

Supplementary Tables

Supplementary Table 1 Database for geometric mean diameter (GMD) and geometric standard deviation (GSD) fitting

No.	Engine type	Altitude	Fuel Type	Fuel flow rate (%)	EI_m Range (mg/kg)	GMD (nm)	GSD	Data Num	Ref.
1	JT8D-219	ground	Jet A	4~100	1.3~260	21~41	1.64~1.79	7	[42]
2	CF6-80A2	ground	Jet A	4~30	0.73~0.45	14.7~17.6	1.46~1.56	4	[42]
3	CF6-80C2B8F	ground	Jet A	4~50	0.26~1.8	15.2~20.6	1.48~1.62	9	[42]
4	PW 2037	ground	Jet A	4~70	1.6~27.4	18.9~31.1	1.58~1.76	7	[42]
5	CFM56-7B24/3	ground	Jet A-1	3~96	1.81~69.3	18.8~40.1*	1.57~1.85*	6	[35]
6	PW4168	ground	Jet A-1	4~99	0.97~77.8	20.2~40.9*	1.59~1.78*	8	[35]
7	CFM56-2-C1	cruise	Jet A	23~38	12.7~82.4	23.5~35.3	1.63~1.86	6	[14]
8	CFM56-2-C1	cruise	HEFA -Jet A	23~38	4.1~37.6	20.9~28.7	1.58~2.03	6	[14]
9$^{\Delta}$	CFM56-2-C1	ground	JP8	4~100	2.4~198.6	15.9~36.9	1.51~1.98	84	[64]

* The GMD and GSD have been corrected in this study by the line loss function in Durdina et al. [65].
$^{\Delta}$ The dataset was only utilized to validate the fitted correlation, not used for the model training.

Supplementary Table 2 Experimental conditions for the dataset

	Engine type	Size distribution	Number instrument	Mass instrument	Probe distance	Line loss correction	Ref.
1	JT8D-219	DMS500 &TSI3071	TSI3022	LII300	Exhaust exit plane	Yes	[42]
2	CF6-80A2	DMS500	TSI3022	LII300	Exhaust exit plane	Yes	[42]
3	CF6-80C2B8F	DMS500	TSI3022	LII300	Exhaust exit plane	Yes	[42]
4	PW 2037	DMS500	TSI3022	LII300	Exhaust exit plane	Yes	[42]
5	CFM56-7B24/3	DMS500	TSI3790E	LII300 &MSS	Exhaust exit plane	No*	[35]
6	PW4168	DMS500	TSI3790E	LII300 &MSS	Exhaust exit plane	No*	[35]
7	CFM56-2-C1	TSI3936	TSI7610 TSI3010	PSAP	30~150 m	Yes	[14]
8	CFM56-2-C1	TSI3936	TSI7610 TSI3010	PSAP	30~150 m	Yes	[14]
9	CFM56-2-C1	SMPS DMS500	TSI3022 TSI3760 TSI3022	PSAP MAAP	1 m	Yes	[64]

* The GMD and GSD were not corrected in the original article, but have been corrected in this study by the line loss function in Durdina et al. [65].

Supplementary Table 3 Coefficients of the regression models

Methods	A_1	A_2	A_3	A_4	A_5	A_6
GMD-Fuel flow rate	20.55	17.42	/	/	/	/
GMD-T_3	155.7	−143.6	51.65	/	/	/
GMD-EI_m	0.8510	7.764	28.16	56.49	/	/
GMD- T_3& EI_m	0.7812	7.289	30.34	85.85	−0.7604	0.5859

Supplementary Table 4 Eight different models for fitting geometric mean diameter (GMD) data

No.	Name	Predictors	Number of parameters	Model form
1	GMD-Fuel flow rate	Fuel flow rate	2 (A_1, A_2)	$GMD = A_1(F_{flow}/F_{flow,\ max}) + A_2$
2	GMD-T_3	T_3	3 (A_1 ~ A_3)	$GMD = A_1(T_3/1000)^2 + A_2(T_3/1000) + A_3$
3	GMD-EI_m	EI_m	4 (A_1 ~ A_4)	$GMD = A_1lg(EI_m)^3 + A_2lg(EI_m)^2 + A_3lg(EIm) + A_4$
4	GMD-T_3&EI_m	T_3, EI_m	6 (A_1 ~ A_6)	$GMD = (A_1lg(EI_m)^3 + A_2lg(EI_m)^2 + A_3lg(EIm) + A_4) \times ((T_3/1000)^2 + A_5(T_3/1000) + A_6)$
5	GMD-T_3&EI_m(4)	T_3, EI_m	7 (A_1 ~ A_7)	$GMD = (A_1lg(EI_m)^4 + A_2lg(EI_m)^3 + A_3lg(EI_m)^2 + A_4lg(EIm) + A_5) \times ((T_3/1000)^2 + A_6(T_3/1000) + A_7)$
6	GMD-T_3&EI_m(5)	T_3, EI_m	8 (A_1 ~ A_8)	$GMD = (A_1lg(EI_m)^5 + A_2lg(EI_m)^4 + A_3lg(EI_m)^3 + A_4lg(EI_m)^2 + A_5lg(EIm) + A_6) \times ((T_3/1000)^2 + A_7(T_3/1000) + A_8)$
7	GMD-T_3&EI_m(6)	T_3, EI_m	9 (A_1 ~ A_9)	$GMD = (A_1lg(EI_m)^6 + A_2lg(EI_m)^5 + A_3lg(EI_m)^4 + A_4lg(EI_m)^3 + A_5lg(EI_m)^2 + A_6lg(EIm) + A_7) \times ((T_3/1000)^2 + A_8(T_3/1000) + A_9)$
8	GMD-T_3&EI_m(7)	T_3, EI_m	10 (A_1 ~ A_{10})	$GMD = (A_1lg(EI_m)^7 + A_2lg(EI_m)^6 + A_3lg(EI_m)^5 + A_4lg(EI_m)^4 + A_5lg(EI_m)^3 + A_6lg(EI_m)^2 + A_7lg(EIm) + A_8) \times ((T_3/1000)^2 + A_9(T_3/1000) + A_{10})$

Supplementary Table 5 Cruise data for the validation of particle number emission estimation

Aircraft	B707-307C	A340-300	ATTAS+	B737-300+	A310-300
Engine Type	PW JT3D-3B	CFM56-5C4	Mk501	CFM56-3B1	CF6-80C2A2
Flight height (100 ft)	328.67<br>(310, 334 342)*	325.4<br>(336.6, 314)	260<br>(260, 260)	260	350
Flight Speed (m s^{-1})	193.33<br>(187, 190, 203)	194.5<br>(195, 194)	156.5<br>(160, 153)	167	180
Fuel flow (kg s^{-1})	0.37	0.26	0.151	0.213	0.4
EI_m (g kg^{-1}-fuel)	0.5	0.01	0.1	0.011	0.019
EI_n (kg^{-1}-fuel)	1.7×10^{15}	1.8×10^{14}	1.7×10^{15}	3.5×10^{14}	6×10^{14}
Measured GMD	/	/	(34, 35)	25	/
Estimated GMD	39.64	26.41	32.38	25.96	28.03
Measured GSD	/	/	1.55	1.55	/
Estimated GSD	1.86	1.68	1.78	1.68	1.71
Reference	[43]	[43]	[70]	[70]	[70]

* The values in the brackets are the measurements during the experiment. Only the means of these data are utilized as the flight condition.
+The particle size distribution for ATTAS and B737-300 aircrafts were measured during SULFUR 6.

Supplementary Table 6 Summary of the black carbon (BC) emission parameters of the global civil aviation at different sub-phases estimated. (Mass: BC mass emissions; Fuel: fuel consumptions; EI_m: fleet average BC mass emission indices; Num: total BC particle emissions; EI_n: fleet average BC number emission indices; GMD: fleet average geometric mean diameter; GSD: fleet average geometric standard deviation. T: Taxi; TO: Take-off; CO: Climb-out; A: Approach; C: Climb; CR: Cruise; D: Descent).

		LTO					CCD				***All***
		T	TO	CO	A	**Total**	C	CR	D	**Total**	
Mass	Value(Gg)	0.11	0.16	0.36	0.094	**0.72**	2.8	5.8	1.6	**8.8**	***9.5***
	Share	1.1%	1.7%	3.7%	1.0%	**7.5**	21.7%	61.0%	1.7%	**92.5%**	***100%***
Fuel	Value(Tg)	9.1	2.5	6.5	4.0	**22.1**	28.7	114.8	14.7	**158.2**	***180.3***
	Share	5.1%	1.4%	3.6%	2.2%	**12.3%**	15.9%	63.7%	8.1%	**87.7%**	***100%***
EI_m	Value(g/kg)	0.012	0.064	0.055	0.023	**0.033**	0.099	0.051	0.011	**0.056**	***0.053***
Num	Mean(10^{25})	0.39	0.13	0.38	0.20	**1.1**	2.6	6.6	0.57	**9.8**	***10.9***
	Std (10^{25})	0.11	0.023	0.079	0.054	**0.22**	0.56	1.3	0.14	**1.9**	***2.1***
	Share	3.6%	1.2%	3.5%	1.9%	**10.2%**	24.0%	60.4%	5.2%	**89.8%**	***100%***
EI_n	Mean(10^{14}/kg)	4.30	5.21	5.94	5.06	**5.02**	9.20	5.75	3.90	**6.20**	***6.06***
	Std (10^{14}/kg)	1.16	0.92	1.21	1.32	**1.00**	1.95	1.16	0.93	**1.22**	***1.18***
GMD	Mean(nm)	24.57	36.96	34.30	27.18	**29.51**	33.66	32.53	24.26	**32.27**	***31.99***
	Std (nm)	0.95	0.94	0.94	1.09	**0.97**	1.11	0.87	0.84	**0.81**	***0.80***
GSD	Mean(-)	1.85	1.81	1.81	1.85	**1.88**	1.84	1.83	1.83	**1.84**	***1.85***
	Std (-)	0.025	0.020	0.023	0.029	**0.023**	0.017	0.017	0.014	**0.016**	***0.016***

Supplementary Table 7 Upper boundaries of each particle size bin (the lowest boundary is 0 nm)

No.	1	2	3	4	5	6	7	8
Size (nm)	10	15.85	25.12	39.81	63.10	100	158.49	251.19
No.	9	10	11	12	13	14	15	16
Size (nm)	251.19	398.11	630.96	1000	1584.89	2500	3981.07	6309.57

Supplementary Table 8 Comparisons between variabilities among the 27 million flights at 7 sub-phases and the uncertainties of the estimated global emissions. The ratio is between uncertainties and variabilities.

	Particle number emission index EIn(BC), (#/kg-fuel)	Geometric mean diameter GMD, (nm)	Geometric standard deviation GSD
Variability	6.92×10^{14}	4.78	0.078
Uncertainty	1.18×10^{14}	0.8	0.016
Ratio	0.17	0.17	0.21

Supplementary Table 9 Comparison between the black carbon (BC) emissions from ECLIPSE V5[53,56] and Bond et al. (2013)[15]

	ECLIPSE V5[53,56]	Bond et al. (2013)[15]
Anthropogenic BC mass (Gg)	7264	4770
Anthropogenic BC number (#)	8.6×10^{27}	6.0×10^{27}
Open burning BC mass (Gg)	\	2760
Open burning BC number (#)	\	1.1×10^{27}

Supplementary Table 10 ANOVA table for the residuals at different altitudes

Source	Sum of squares (SS)	Degree of freedom (df)	Mean squares (MS)	*F*-statistic	*p* value
Groups	5.60	4	1.40	0.5	0.73
Error	47.24	17	2.78		
Total	52.84	21			

Supplementary Table 11 The main input data required by our black carbon (BC) number emission estimation method.

Estimated parameters	Input data		
	Flight data[40,67]	Engine Specific Data [19]	BC particle parameters[39]
EI_n	Flight altitude	Maximum fuel flow rate	Prefactor *C*
GMD	Fuel flow rate	π_{00} (Engine pressure ratio)	Exponent ε
GSD	Flight speed		
	Mass emission indices		

Supplementary Note 1. Data preparation

A database has been prepared as shown in Supplementary Table 1 to investigate the correlation between the GMD/GSD of BC particles and the engine parameters. Most of the data are measured by utilizing the standardized system, e.g. the Missouri University of Science and Technology (Missouri S&T) system [42] and the Swiss Federal Laboratories for Materials Science and Technology (EMPA) system [35]. The NASA data [14] are not measured by the standard system, but they are currently the most comprehensive measurements at cruise conditions. The influences of alternative fuels are also included. As a result, the NASA data are included. The summary of the conditions for these experiments are shown in Supplementary Table 2.

There are 53 available datasets, including 7 types of aircraft engines from different manufacturers to cover a wide range of BC emissions: JT8D-219, PW2037, PW4168 (Pratt & Whitney Aircraft Group), CF6-80A2, CF6-80A2 (General Electric), CFM56-7B24/3, CFM56-2-C1 (CFM International). The fuel flow rate, EI_m, GMD and GSD are simultaneously measured. The EI_m ranges from 0.26 to 260 mg per kg-fuel with the thrust levels between 3% and 100%. The GMDs are from 14.7 to 40.9 nm and the GSDs locate between 1.46 and 2.03.

Supplementary Note 2. GMD-Fuel flow rate correlation

Most of the emission data are measured on the ground. The measurements of GMD or GSD are commonly assumed to be a linear function of the thrust settings[37]. The ratios of fuel flow rates ($\dot{m}_f / \dot{m}_{f,\max}$) and thrusts ($F / F_{OO}$) are usually used interchangeably[26,27], where $\dot{m}_f$ and $\dot{m}_{f,\max}$ are respectively the measured and maximum fuel flow rates, F and F_{OO} are respectively the measured thrust and the maximum rated thrust. Supplementary Figure 1 (a) shows the measured GMD versus the ratio of fuel flow rates which is represented by a linear function in equation (S.1)

$$GMD = A_1 \cdot \left(\frac{F_{flow}}{F_{flow,\max}} \right) + A_2 \,. \qquad \text{(S.1)}$$

The least square linear regression was utilized to estimate the parameters. However, the data measured at cruise conditions (solid dots) are not in line with the data measured on the ground (open dots). Teoh et al.[37] adopted two different linear functions to respectively fit the relations at cruise and ground conditions. But the fitted relation at cruise conditions suffers from limited data. The relation for the ground data also has high uncertainty, as evidenced by the highly scattered data. Recent studies [65] showed that the linear relation between the fuel and thrust is only valid at Sea Level Standard (SLS) conditions. The relation becomes nonlinear with the increasing altitude, so the fuel flow and the thrust rates are no longer interchangeable. It becomes difficult to obtain the thrust at cruise conditions, leading to additional difficulties to fit the GMD as a linear function of thrust.

Supplementary Note 3. GMD-T_3 correlation

The abundant ground measurements can be utilized to estimate the particle size at cruise, if the GMDs at both ground and cruise conditions follow a generic predictive relation. Durdina et al. [65] assumed that the GMD is solely a function of the combustor inlet temperature (T_3) at all flight conditions, which is supported by a series of experiments conducted in a test cell with simulated flight altitudes from sea level to 15.2 km[63].

In the test experiments, the BC particle size distributions were measured for a typical turbofan engine operated at different simulated altitudes (from sea-level-static to 15.2 km, about 0.8 Mach) in a test facility at Arnold Engineering Development Center (AEDC)[63]. The dataset showed that the size distribution of BC particles was strongly dependent on the engine inlet temperature T_3. The fitted relation between the particle size and T_3 is applicable for different simulated altitudes inside the test facility.

The detailed results are shown in Supplementary Figure 15. The GMDs of the size distributions for soot particles were extracted and calculated by Durdina et al. [65]. A quadratic equation was utilized to fit the data at different simulated altitudes, which is similar to the GMD-T_3 relation in Supplementary Figure 1. It is shown that all the data are in a good agreement with the fitted line, in spite of the different operating conditions and the altitudes.

It is notable that the sea-level-static condition (blue dots) and the 12.2 km 0.8 Mach condition (yellow dots) have the similar tested combustor inlet temperatures T_3 between 500 K and 750 K. The measured particle sizes of these two conditions were also quite comparable to each other, both with GMDs between 25 and 35 nm. The residuals between the measurements and the fitted relation were calculated for all the different altitudes as shown in the right panel of Supplementary Figure 15. There is no significant correlation between the residuals and the altitudes.

The analysis of variance (ANOVA) test was conducted to investigate the differences among the residuals at different altitudes, which indicates that there is no statistically significant difference among them. The ANOVA table is shown in Supplementary Table 10, with a p value of 0.73, which means there is a high risk (73%) to claim that the residuals are different. As a result, the experimental results indicate that the dependence of BC particle size on T_3 is applicable to different simulated altitudes.

In the present study, the T_3 temperature is estimated following the method in FOX method[26], which is briefly described in Supplementary Note 18. Supplementary Figure 1(b) shows the relation between GMDs and estimated T_3 temperatures. There is no distinct difference between the cruise and ground data, therefore a quadratic equation (S.2)

$$GMD = A_1 \cdot \left(\frac{T_3}{1000} \right)^2 + A_2 \cdot \left(\frac{T_3}{1000} \right) + A_3, \tag{S.2}$$

is adopted to fit the data. All the data are within the prediction interval of 95% confidence. The least square linear regression was utilized to estimate the parameters.

In Supplementary Figure 16, the data at different simulated altitudes were compared with the data utilized to develop the GMD-T_3 correlation. The results indicate that new dataset (colorful dots) agrees well with the utilized dataset (grey dots). All of the new data are within 95% prediction interval of the fitted correlation.

Supplementary Note 4. GMD-EI_m correlation

The mass emission index EI_m is also a potential variable of a universal fitting relation for the GMDs at different flight conditions, as suggested by Brem et al.[34]. The relation between the GMD and EI_m is shown in Supplementary Figure 1(c). Similar to the GMD-T_3 relation, all the data at cruise (solid dots) and ground (open dots) follow the same trend. A cubic equation (S.3) is adopted to fit the data

$$GMD = A_1 \cdot \lg\left(EI_m\right)^3 + A_2 \cdot \lg\left(EI_m\right)^2 + A_3 \cdot \lg\left(EI_m\right) + A_4 \ . \tag{S.3}$$

The least square linear regression was utilized to estimate the parameters. Two statistical metrics, adjusted R^2, and root mean square deviation (RMSE), are utilized to evaluate goodness of the fitting results. The adjusted R^2 was utilized the influence of the number of parameters in the fitted relation.

Supplementary Note 5. GMD-T_3&EI_m correlation

The performances of the above three different fitted relations are compared in the bottom row of Supplementary Figure 1. The often adopted GMD-fuel flow rate (thrust) relation has the lowest quality, with a high RMSE (4.03) and a relatively low adjusted R^2 (0.63). The RMSEs respectively decreased by 8.8% and 30.0% when using the GMD-T_3 and GMD-EI_m relations. The adjusted R^2 are also improved by 7.9% and 28.6%.

Although the qualities of the fitted correlations have been improved based on T_3 and EI_m, the data scattering is still significant, which indicates that single type input, such as the fuel flow rate, thrust, or T_3 is insufficient to adequately predict the GMD. Therefore, we try to develop the correlation for GMD as a phenomenological model, not based on understanding of the causal relationship between the engine parameters and the exhaust characteristics. We observe that both GMD-T_3 and GMD-EI_m relations are independent of the flight conditions and they are mutually complementary, i.e. GMD values of certain engines (e.g. CF6-80C2B8F, CF6-80A2) are overestimated based on the GMD-T_3 correlation, but underestimated when EI_m is used. Consequently, we develop a new correlation using both T_3 and EI_m as input parameters (equation (S.4)) in an attempt to improve the quality of the predictive model.

The Levenberg-Marquardt nonlinear least squares algorithm, which can be viewed as a combined algorithm of Gauss-Newton and gradient descent methods, was used to iteratively minimize the sum of the squares of the deviations between the observations and the predicted values. We utilized the function "nlinfit" in the Statistics and Machine Learning Toolbox of MATLAB (Version 2015a) to estimate the parameters. The initial value for the iterative algorithm was [1 1 1 1 1 1], and the final estimated coefficients were [0.7812 7.289 30.34 85.85 -0.7605 0.5859]. Termination tolerance on the estimated coefficients was 10^{-8}.

$$GMD = \left(A_1 \cdot \left(\lg\left(EI_m\right)\right)^3 + A_2 \cdot \left(\lg\left(EI_m\right)\right)^2 + A_3 \cdot \lg\left(EI_m\right) + A_4\right) \cdot \left(\left(\frac{T_3}{1000}\right)^2 + A_5 \cdot \frac{T_3}{1000} + A_6\right) \quad \text{(S.4)}$$

The improvement of the new correlation is discussed in the main article.

The coefficients of all the fitted relations are shown in Supplementary Table 3. As shown in the main body, the quality of the GMD-T_3&EI_m correlation outperforms the others, as evidenced by the higher

adjusted R^2 and the lower RMSE. The bias-variance trade-off tests were also conducted to investigate whether the GMD-T_3&EI_m correlation was over-fitted in Supplementary Note 6.

Supplementary Note 6. Bias-variance trade-off tests

In order to investigate the bias-variance trade-off of our models, all the data of measured particle size distributions were randomly divided into the training (33 data points about 60%) and validation (20 data points about 40%) sets. One thousand stochastic groupings were generated and calculated for the bias-variance trade-off tests to avoid the influence of the grouping method. Both the training and validation datasets contained the data from all the engines.

Eight different models were utilized as shown in Supplementary Table 4. The model complexity increased with the number of parameters ranging from 2 to 10. Models No.1 to No.4 were discussed in the main body, and model No. 4 was the one we used for calculation in our study. Models No.5 to No.8 were only used here to investigate the bias-variance trade-off. Only the complexity in the EI_m term was increased by varying the polynomial order from 4 to 7. The model performance on the training dataset would substantially deteriorate if we also increased the complexity of the T_3 term, which was caused by the quadratic dependence of GMD on T_3.

The models were only fitted based on the training dataset. Then the fitted relations were applied to the validation dataset to test the model quality, which was quantified by the root-mean-square errors between the model results and the data. Supplementary Figure 2 shows the results of the bias-variance trade-off tests. The shaded area indicated the uncertainties obtained from 1000 different groupings. Both the training and the validation errors were high, when using the less complex models, e.g. GMD-Fuel flow rate, indicating the bias was large. When we increased the model complexity, the training and validation errors first simultaneously decreased, and then the training errors remained nearly constant with a slight drop, while the validation errors increased. The

uncertainties of the validation errors became very large when more complicated models were utilized, which indicated that the model performance may be extremely poor for some situations. The variance became high for the models with high validation errors and low training errors, which means the models were over-fitted. The GMD-T_3&EI_m relation was near the turning point of the validation error curve. This model achieves the balance between bias and variance, so it was the optimal model based on the current dataset.

Supplementary Note 7. Independent validation using APEX dataset

In addition to the bias-variance trade-off tests, a new completely independent dataset is also included to investigate the model performance. The dataset is from Aircraft Particle Emissions eXperiment (APEX) [64], containing 84 data points measured for 11 power settings from 4% to 100%. The APEX experiments measured the particle emissions from NASA's DC-8 aircraft, equipped with four General Electric CFM56-2-C1 engines. The system utilized a heated sampling channel (300 ℃) to remove volatile components and the line loss corrections were conducted. The BC particle mass, number and size distributions were simultaneously measured. Here, we only used the data for standard JP-8 fuel sampled 1 m behind the engine exit, where the non-volatile particles dominated the total emissions. The particle size distributions were measured by NASA Scanning Mobility Particle Sizing (SMPS) and Differential Mobility Spectrometer (DMS-500). The summary of the APEX data is shown in Supplementary Table 1.

The estimated GMD, GSD and EI_n(BC) using our model are compared with the APEX data which are not included in the model training. Supplementary Figure 3 shows the comparisons. For the GMDs, the model results have a good correlation (0.91) with the measurements. The model generally overestimates the data with a RMSE of 3.33. For GSDs, the results are similar with the model training results shown in Figure 1 of the main body. The estimated particle number emission has a good agreement with the measurements, similar to those in Figure 2 of the main body.

It should be noted that the measurement uncertainties (horizontal error bars) are large. Most of the data utilized for the model training are from Aviation-Particle Regulatory Instrumentation Demonstration Experiment (A-PRIDE), which used a different system from the NASA APEX experiments. The results are reasonable considering the measurement uncertainties and the different measurement systems.

Through the bias-variance trade-off tests and comparison with the independent APEX dataset, we demonstrate that our model does not over-fit the data.

Supplementary Note 8. GSD estimation

The Geometric Standard Deviation (GSD) of the BC particles is also an important parameter for the particle number emission estimation. The relations between GSD and EI_m, T_3 are fitted. Different from the situation for GMD, the dependence of GSD on T_3 is not strong enough, as shown in Supplementary Figure 4 (a). The data point has large scattering around the fitted line. The correlation between GSD and EI_m is much stronger. A simple linear relation between the GSD and the EI_m is adopted to predict the GSD in this study, as shown in Supplementary Figure 4 (b). The variation of GSD is not as large as that of GMD. As a result, the fitted GSD-EI_m correlation is adopted in this study.

Supplementary Note 9. The uncertainties of particle mass parameters

The uncertainties of ε shown in Abegglen et al.[39] were the 95% confidence interval, not the standard deviation. Besides, the uncertainties were significantly overestimated. In Abegglen et al. [39], ε was estimated by fitting the power-law relationship between particle mass and size

$$m_p = C \cdot d_m^{\varepsilon}, \qquad \text{(S.5)}$$

The particle mass was directly measured by Centrifugal Particle Mass Analyzer (CPMA;

Cambustion Ltd., Cambridge, UK) at fixed particle sizes and engine thrusts. Based on the measurements, the standard deviations for the mass of the size- and thrust-selected particles were maximum 16.6% for the particles smaller than 40 nm and 11.0% for those larger than 40 nm. We re-analyzed the data in the reference to determine the uncertainties of the mass data more accurately.

Supplementary Figure 5 shows the measured particle mass and uncertainties (error bars) for 33%, 67% and 105% thrusts from Abegglen et al. [39]. The particle sizes are between 20 to 150 nm. The mass uncertainties calculated using the ε data in Abegglen et al.[39] are represented by the dashed lines, which are far from the measured data showing that the uncertainties are dramatically overestimated. The shaded area is the results estimated using an uncertainty of 0.025 for ε. The estimated uncertainties are close to those of the measurements.

According to Abegglen et al. [39], the variation of prefactor C is very small, within about 5%. As a result, for a given particle size (d_m), the change of mass caused by the parameter uncertainties is

$$\frac{m_p}{E\left(m_p\right)} = \frac{C}{E(C)} d_m^{\varepsilon - E(\varepsilon)} \approx d_m^{\Delta\varepsilon}, \qquad \text{(S.6)}$$

where $E(m_p)$ and $E(\varepsilon)$ are the means of particle mass and ε. For the particles with an electrical mobility diameter (d_m) between 20 to 150 nm, the changes of particle mass are about ±40% with the ε uncertainty utilized in this study ($\Delta\varepsilon$=0.025). These estimated particle mass uncertainties are already larger than the measurements (11.0% to 16.6%). We assume that it is reasonable to use larger uncertainties for particle mass considering the measurements are only for one type of engine. However, the changes of particle mass can be as large as -90% to 2000% if the uncertainties of ε (0.04~0.3) in Abegglen et al. [39] are utilized. The values are far away from the measurements, leading to unrealistic results. As a result, the standard deviation of ε was assumed to be 0.025.

Supplementary Note 10. Residuals of the EI_n estimation

Supplementary Figure 6 shows the residuals of all the Monte Carlo runs separately for the entire data (cruise + ground), cruise and ground measurement data. The residuals are close to normal distributions. The mean of the residual for all the data is 0.84×10^{13}/kg-fuel with a 95% confidence interval between $(-0.16\ 1.84)\times10^{13}$. The mean residual is small compared to the BC particle number emission index which is normally at the magnitude of 10^{14}/kg-fuel. The standard deviation is 1.43×10^{14}/kg-fuel, the same magnitude with the emission index.

The EI_n data for the cruise and ground conditions are respectively over- and underestimated, evidenced by the mean residuals of 7.82×10^{13} and -1.39×10^{13}/kg-fuel. The bias may be caused by both the experimental and modeling uncertainties, e.g. the flight parameters, atmospheric conditions and measurement technologies. The limited number of data points may also influence the results. The biases are within the uncertainties of the global mean EI_n(BC) estimations, which have the standard deviations of 1.00×10^{14}/kg-fuel for LTO and 1.22×10^{14}/kg-fuel for CCD as shown in Supplementary Table 6.

Supplementary Note 11. Sensitivity tests for the influences of BC mass emissions

The BC mass emission directly influences the estimation of particle number emission. Sensitivity tests were conducted to investigate the influence of BC mass emissions on the particle number emission and the size distribution. The estimations based on the EEA dataset were utilized as the baseline in the sensitivity tests. Besides the baseline, four different cases with substantial changes in BC mass emissions were included. It was assumed that the BC mass emissions were respectively 33% (1/3, 3.2 Gg), 50% (1/2, 4.8 Gg), 150% (14.3 Gg) and 200% (19.1 Gg) of the baseline emission (9.5 Gg). The range of these values is able to cover most of the BC mass emissions in the literature, as shown in Table 2.

Supplementary Figure 9 shows the results of the sensitivity tests. The changes of particle number emissions (Supplementary Figure 9 a) are much smaller than the corresponding changes of mass emissions, because higher $EI_m(BC)$ induces larger and heavier particles, which alleviates the increase of BC particle number and makes the particle number emission relatively stable. It is similar when $EI_m(BC)$ is lower with smaller and lighter particles. Supplementary Figure 9 a) shows that, for the cases with 50% and 150% mass emissions, the resulted particle number emissions are respectively 7.7×10^{25} and 13.4×10^{25}, and the deviations from the baseline estimation (10.9×10^{25}) are about 1.5 and 1.2 times of the standard deviation of baseline, σ_{num} (2.1×10^{25}). Even with the largest changes (33% and 200%) in BC mass emissions, the variations of the estimated particle number emissions (6.0×10^{25} and 15.5×10^{25}) are still near two times of σ_{num}, which are indicated as the dashed lines. Generally, the number emission is more sensitive when the BC mass emission is lower.

The GMDs of all the emitted particles are 30.04 and 33.37 nm for the cases with 50% and 150% BC mass emissions. The changes are respectively −1.95 and 1.38 nm from the baseline (31.99nm), about −2.4 and 1.7 times of the estimated standard deviation σ_{gmd} (0.8 nm). For the 200% case, the change of GMD (2.37 nm) is still within $3\sigma_{gmd}$. For the 33% case, deviation from the baseline is stronger with the change of GMD −3.1 nm, beyond $3\sigma_{gmd}$. The GMD is more sensitive to the change of BC mass emission than the particle number emission.

The changes of GSDs are quite small. As shown in Supplementary Figure 9 c), nearly all the changes of GSDs are still within 2 times of the estimated GSD standard deviation σ_{gsd} (0.016), except the 33% case. For the 200% case, the GSD (1.87) is increased by 0.02 relative to the baseline (1.85) and the change is only about 1.25 times of σ_{gsd}. For the 50% case, the GSD is 1.82, with a difference of $-1.88\sigma_{gsd}$ from the baseline.

In summary, the sensitivity tests indicate that our estimations of the BC number emission and size distribution were robust, even when we applied substantial changes of the BC mass emission from 33% to 200% of the baseline. The sensitivity tests demonstrate that the BC particle number emission and size distribution are rather robust, considering the substantial changes of the BC mass emissions used in the tests. Therefore we consider the uncertainties are at reasonable based on the results.

Supplementary Note 12. Monte Carlo simulations and uncertainty estimations

By Monte Carlo (MC) Simulations, different total emissions were obtained based on an ensemble of the uncertain parameters (GMD, GSD and particle mass parameters of different engines) utilized to convert mass into number emissions, as shown in Supplementary Figure 12. The global BC size distribution was obtained by 100 MC simulations. The number of MC runs is sufficient to reach steady state as shown in Supplementary Figure 11.

In order to estimate the uncertainties, we first integrated the global distributions of all the aircraft BC emissions from millions of flights as shown in Supplementary Figure 12. The emitted particles from each aircraft were divided into 16 size bins as shown in Supplementary Table 7. The global inventory with 16 size bins was calculated by integrating the size-resolved emissions from all the aircrafts. Namely, we summed all the distributions for every aircraft over a full year. Finally, the uncertainties were calculated based on the MC results to quantify the reliability of these overall statistics (GMD, GSD and EI_n).

The uncertainties of the estimations are not the same concept as the “variabilities” among different engines and flight conditions as shown in Supplementary Figure 12. The variabilities are usually calculated as the standard deviations of the corresponding parameters from all the aircrafts. In this study, the global GMD and GSD were obtained by fitting the distribution of the BC particles emitted from all the aircrafts during one year, and they were not simply the mean of the values of all the

aircrafts. The uncertainties of EI_ns, GMDs and GSDs were not simply the standard deviation of values of all the aircrafts. Instead, we assessed the uncertainties of our estimations induced by the utilized uncertain parameters based on Monte Carlo (MC) Simulations, as shown in Supplementary Figure 12. As a result, the uncertainties here do not represent the variabilities among different flights, but they indicate the influence of the uncertain parameters on the estimations.

The mean and standard deviation from the MC runs are shown in Figure 4. The GMD and GSD of the global BC size distribution were obtained by fitting the particle numbers in the size bins into the lognormal distribution for each MC run, and the uncertainties of GMD and GSD were estimated from these MC runs.

In Supplementary Table 8, the variabilities were calculated as the standard deviations of the emissions from all the 27 million flights at the seven sub-phases (taxi, take-off, climb-out, approach, climb, cruise and descent). The uncertainties are about 17% to 21% of the variabilities.

Supplementary Note 13. Estimation of BC particle number emission from open burning

The BC particle number emission from open burning is not directly available in the literature. In a comprehensive review, Bond et al. [15] reported that the BC mass emission of open burning was about 2760 Gg per year. In this study, we roughly estimate the BC particle number emission by converting the mass emission into number emission, assuming a lognormal distribution of the BC particles and utilizing an effective density of 10^3 kg m^{-3}. The parameters for the lognormal distributions of urban emissions and open burning emissions were obtained from the data in Bond et al. [15] as shown in Supplementary Figure 14. The GMDs were 75.2nm and 140.6nm respectively for the urban and open burning emissions. The BC particles from open burning are normally larger than those from urban emissions, which mainly originate from energy-related applications which have higher combustion efficiencies.

The mass to number conversion follows the similar methods for the aviation emissions. Assuming that the particle is spherical, we can express the particle mass m_p as

$$m_p = \rho \cdot \frac{1}{6}\pi \cdot d^3, \qquad \text{(S.7)}$$

where ρ is the effective density of the BC particle, which is assumed to be 10^3 kg m^{-3}; d is the volume equivalent diameter, which follows the lognormal distribution as shown in Supplementary Figure 14. The total BC mass *Mass* can be calculated as

$$Mass = \int_0^\infty m_p \cdot Num \cdot n(d_m)\,\mathrm{d}d_m = \rho \cdot \frac{1}{6}\pi \cdot Num \int_0^\infty d^3 \cdot n(d_m)\,\mathrm{d}d_m, \qquad \text{(S.8)}$$

where *Num* is the total number of the BC particles. According to the properties of lognormal distribution (Equation (S.17)) described in Supplementary Note 15, the total BC mass is calculated as

$$Mass = \rho \cdot \frac{1}{6}\pi \cdot Num \cdot GMD^3 \cdot \exp\left(\frac{3^2 \cdot (\ln(GSD))^2}{2}\right). \qquad \text{(S.9)}$$

The GMD and GSD are the parameters of the lognormal distributions shown in Supplementary Figure 14. The total BC particle number emission can be estimated as

$$Num = \frac{Mass}{\rho \cdot \frac{1}{6}\pi \cdot GMD^3 \cdot \exp\left(\frac{3^2 \cdot (\ln(GSD))^2}{2}\right)}. \qquad \text{(S.10)}$$

The estimated BC particle number emission of open burning per year is about 1.1×10^{27} as shown in Supplementary Table 9.

In order to evaluate the validity of this rough estimation method, we used the method to calculate the BC number emission based on the total BC mass emission (7264 Gg per year) in ECLIPSE V5 BC inventory[56], and the result was 9.1×10^{27} per year which was close to the estimation (8.6×10^{27} per year) by Paasonen et al.[53] using the same mass inventory.

In this study, we adopted the global anthropogenic BC mass emission inventory ECLIPSE V5[56] to compare with the aviation emissions. The ECLIPSE inventory was utilized as a reference for energy-related emissions by Bond et al. (2013)[15], but the value in Version 5 was higher than that reported in Bond et al. (2013) (See Supplementary Table 9), which was mainly due to the inclusion of emissions from kerosene wick lamps, gas flaring, and re-estimation of the emissions from China using the regional coal statistics[56].

Supplementary Note 14. Power law between particle mass and diameter

Soot particles show self-similarity on different scales, so the fractal scaling law can be utilized to relate the fractal aggregate size and the number of primary particles (n_{pp})[39,60,61].

$$n_{pp} = k\left(\frac{d_m}{d_{pp}}\right)^{D_m} \quad \text{(S.11)}$$

where d_{pp} is the volume area equivalent primary particle diameter, d_m is the mobility diameter of an aggregate, k is a scaling prefactor and D_m is the mass mobility exponent.

According to TEM measurements[7], the primary BC particles were nearly spherical. The mass of the aggregate particle can be calculated as

$$m_p = n_{pp}\cdot\left(\rho_0\cdot\frac{\pi}{6}\cdot d_{pp}^3\right) = k\left(\frac{d_m}{d_{pp}}\right)^{D_m}\cdot\left(\rho_0\cdot\frac{\pi}{6}\cdot d_{pp}^3\right) = \frac{\pi}{6}\rho_0\cdot k\cdot d_m^{D_m}\cdot d_{pp}^{3-D_m}, \quad \text{(S.12)}$$

where ρ_0 is the density of primary particles. According to the FA theory[71] and the recent measurements of jet engine[36], the primary particle diameter and mobility diameter of an aggregate has the following relation:

$$d_{pp} = f\cdot d_m^{\xi}, \quad \text{(S.13)}$$

where f and ξ are constant parameters. The mass of aggregates is expressed as

$$m_p = \frac{\pi}{6}\rho_0 \cdot k \cdot d_m^{D_m} \cdot \left(f \cdot d_m^{\xi}\right)^{3-D_m} = \left(\frac{\pi}{6}\rho_0 \cdot k \cdot f^{3-D_m}\right) \cdot d_m^{\left((1-\xi)D_m+3\xi\right)} = C \cdot d_m^{\varepsilon}. \quad \text{(S.14)}$$

The prefactor contains the density of the primary particle. However, Abegglen et al. [39] did not assume any density. They directly measured the particle mass with desired sizes selected by a Differential Mobility Analyzer (DMA) under specific thrusts. The ε and prefactor C were estimated by fitting the power-law relationship between particle mass and size. It is difficult to estimate ρ_0 from the prefactor C, because the parameters k and f were not specifically estimated.

Supplementary Note 15. Particle mass-number conversion

The particle mass emission index can be expressed as the integration of all the mass of the single particles:

$$EI_m = \int_0^\infty m_p \cdot EI_n \cdot n\left(d_m\right) \mathrm{d}d_m \quad \text{(S.15)}$$

$$EI_m = \int_0^\infty C \cdot d_m^{\varepsilon} \cdot EI_n \cdot n\left(d_m\right) \mathrm{d}d_m = C \cdot EI_n \cdot \int_0^\infty d_m^{\varepsilon} \cdot n\left(d_m\right) \mathrm{d}d_m \quad \text{(S.16)}$$

We first introduce a theorem: according to the properties of lognormal distribution[72], if a random variable X follows the lognormal distribution with parameters μ and σ, for any real number t we have:

$$E\left(X^t\right) = \exp\left(\mu t + \frac{1}{2}\sigma^2 t^2\right), \quad \text{(S.17)}$$

Since d_m follows the lognormal distribution $n(d_m)$, we have

$$EI_m = C \cdot EI_n \cdot \int_0^\infty d_m^{\varepsilon} \cdot n\left(d_m\right) \mathrm{d}d_m = C \cdot EI_n \cdot E\left(d^{\varepsilon}\right) = C \cdot EI_n \cdot \exp\left(\mu\varepsilon + \frac{1}{2}\sigma^2\varepsilon^2\right). \quad \text{(S.18)}$$

According to the definitions of GMD and GSD, there are

$$GMD = \exp\left(\mu\right), \quad \text{(S.19)}$$

$$GSD = \exp(\sigma), \quad \text{(S.20)}$$

Finally, the relation between EI_m and EI_n is expressed as

$$EI_m = C \cdot EI_n \cdot \left(\exp\left(\mu\right)\right)^{\varepsilon} \cdot \exp\left(\frac{1}{2}\sigma^2\varepsilon^2\right) = C \cdot EI_n \cdot GMD^{\varepsilon} \cdot \exp\left(\frac{\varepsilon^2 \cdot \left(\ln\left(GSD\right)\right)^2}{2}\right). \quad \text{(S.21)}$$

Supplementary Note 16. Independent validation using an engine with a double annular combustor

In order to investigate the performance of the proposed method for different engine designs, the estimations are compared with a new dataset[36] from a jet engine with a double annular combustor (CFM56-5B4-2P), which is a special engine, not widely used in the current fleets. There are one outer (pilot) annulus and one inner (main) annulus in this engine. For low thrusts, only the pilot annulus is fueled. For high thrusts, the main annulus is also triggered. As a result, the operating conditions are totally different from the engines with a single annular combustor utilized to develop our correlation.

Supplementary Figure 17 a) shows the changes of the GMD with the thrust. It shows that the estimations (solid line) follow the measurements (red dots). All the measurements are within the 95% confidence prediction interval of the estimations. Before the start of the main annulus, the GMD increases with the thrust from about 20 nm to about 30 nm. The particle size greatly drops from 30 nm to about 20 nm after the start of the main annulus, because the utilization of the main annulus enhances the oxidation of the generated BC particles, leading to a much lower BC mass emission. As a result, the GMD becomes much smaller according to the proposed correlation Equation (6) in this study. There is a slight increase in GMD from about 25% to 100% thrust, due to the increase of T_3. The correlation coefficient (*r*) between the estimated GMDs and the measurements is as high as 0.9 as shown in Supplementary Figure 17 b).

The comparisons between the estimated BC particle number emissions and the measurements are shown in Supplementary Figure 17 c) and d). The proposed relation captures the changes of EI_n(BC) with the thrust reasonably well. For the low thrusts, the predictions are generally underestimated, but

most of the measurements are still within a factor of two of the estimations. For high thrusts after the start of the main annulus, the estimations are in a better agreement with the measured data. Nearly all the data are within the standard deviation of the predictions. The overall correlation coefficient (*r*) between the predictions and measurements is 0.94. The main statistical metrics are comparable with those from the engines with a single annular combustor as shown in Figure 2. The GSD data are not fully available for the double annular engine. Only the data for 6%, 17% and 31% of the maximum thrust are available, so we only compared the three data points with the data which were utilized to develop the GSD-EI_m(BC) correlation and the fitted relation as shown in Supplementary Figure 18. The new data (red dots) agree well with the utilized dataset (grey dots). All of the new data are within 95% prediction interval of the fitted correlation. At 31% thrust, the main annulus started, leading to lower BC mass emission. The measured GSD at 31% is smaller than that at 17%, which indicates a strong relation between GSD and EI_m(BC).

In general, the proposed method successfully captures the unique changes of GMD of the BC particles generated in this double annular engine, even though the method is developed based on the engines with a single annular combustor. The results demonstrate the necessities to include both the T_3 and EI_m(BC) as the predictors for GMDs. They also show the potential abilities of the proposed method to estimate the BC particle size distribution and the number emission for significantly different engine designs and operating conditions.

Supplementary Note 17. R^2 for the nonlinear regression of GMD-T_3&EI_m correlation

Normally, the adjusted R^2 is applicable to the linear fittings. Here we show that it is also valid for the current GMD-T_3&EI_m correlation. In order to calculate an effective R^2

$$R^2 = 1 - \frac{Err_{res}}{Err_{tot}} = \frac{Err_{reg}}{Err_{tot}}, \tag{S.22}$$

we should have

$$Err_{tot} = Err_{reg} + Err_{res}\,, \tag{S.23}$$

where Err_{tot} is variance of the data y_i; Err_{reg} is the explainable variance by the regression f_i; Err_{res} is the residual or unexplainable variance:

$$Err_{tot} = \sum_{i=1}^{n}\left(y_i - \bar{y}\right)^2; Err_{reg} = \sum_{i=1}^{n}\left(f_i - \bar{y}\right)^2; Err_{res} = \sum_{i=1}^{n}\left(y_i - f_i\right)^2\,. \tag{S.24}$$

Based on the definition of Err_{tot}, we can have

$$\begin{aligned} Err_{tot} &= \sum_{i=1}^{n}\left(y_i - \bar{y}\right)^2 = \sum_{i=1}^{n}\left(y_i - f_i\right)^2 + \sum_{i=1}^{n}\left(f_i - \bar{y}\right)^2 + 2\sum_{i=1}^{n}\left(\left(y_i - f_i\right)\left(f_i - \bar{y}\right)\right) \\ &= Err_{res} + Err_{reg} + 2\sum_{i=1}^{n}\left(\left(y_i - f_i\right)\left(f_i - \bar{y}\right)\right) \end{aligned} \tag{S.25}$$

Equation (S.23) can be proved, if the third term is zero.

$$\sum_{i=1}^{n}\left(\left(y_i - f_i\right)\left(f_i - \bar{y}\right)\right) = \sum_{i=1}^{n}\left(y_i f_i\right) - \sum_{i=1}^{n}\left(f_i f_i\right) - \sum_{i=1}^{n}\left(y_i \bar{y}\right) + \sum_{i=1}^{n}\left(f_i \bar{y}\right) \tag{S.26}$$

Here, we suppose a new function *g(a, b)*:

$$g\left(a,b\right) = \sum_{i=1}^{n}\left(a \cdot f_i + b - y_i\right)^2 \tag{S.27}$$

It is necessary to introduce two assumptions: (1) the cost function is in the form of least square; (2) scaling (multiplied by a) or shifting (added by b) of the model *f* would not improve the fitting. If the two assumptions are satisfied, then $\sum_{i=1}^{n}\left(f_i - y_i\right)^2$ is the optimized fitting, namely the function *g(a, b)* can achieve the minimum value when a=1 and b=0. Then we have

$$\frac{\partial g\left(a,b\right)}{\partial a}\Big|_{a=1,b=0} = \frac{\partial g\left(a,b\right)}{\partial b}\Big|_{a=1,b=0} = 0 \tag{S.28}$$

$$\begin{aligned} \frac{\partial g\left(a,b\right)}{\partial a}\Big|_{a=1,b=0} &= 2\sum_{i=1}^{n}\left(\left(f_i - y_i\right) f_i\right) = 0 \\ \frac{\partial g\left(a,b\right)}{\partial b}\Big|_{a=1,b=0} &= 2\sum_{i=1}^{n}\left(f_i - y_i\right) = 0 \end{aligned} \tag{S.29}$$

As a result, we have

$$\sum_{i=1}^{n}\left(\left(y_i - f_i\right)\left(f_i - \bar{y}\right)\right) = 0 \tag{S.30}$$

Then Equation (S.23) is proved.

For the linear regressions with a constant term, the two assumptions can be satisfied. Equation (6) meets the first assumption, because the Levenberg-Marquardt nonlinear least squares algorithm was utilized. For the second assumption, scaling (multiplied by a) of the model *f* cannot improve the fitting, and shifting (added by b) of the model *f* also barely changes the results, because there is a constant term $A_4 \cdot A_6$. As a result, R^2 is still applicable for the nonlinear regression of GMD-T_3&EI_m correlation.

Supplementary Note 18. T_3 estimation

The T_3 temperature is estimated following FOX method[26]. The temperature at the ground condition can be estimated as:

$$T_3 = T_2 \cdot \left(\frac{P_3}{P_2}\right)^{\frac{\gamma-1}{\gamma \cdot \eta_P}} \tag{S.31}$$

$$P_3 \approx \left(\pi_{00} - 1\right) \cdot \left(\frac{\dot{m}_f}{\dot{m}_{f,\max}}\right) + 1 \tag{S.32}$$

where P_3 is the combustor inlet pressure with the unit of atm, π_{00} is the overall pressure ratio, T_2 and P_2 are respectively the engine inlet temperature and pressure, namely the ambient temperature and pressure during the ground experiments, γ is the heat capacity ratio (γ = 1.4) and η_P is the polytropic efficiency which is assumed as 0.9[26].

For the cruise condition, the engine inlet temperature and pressure have to be revised based on the speed of aircraft (Mach number, Ma) and the ambient pressure and temperature at cruise (P_s, T_s):

$$P_2 = P_s \cdot \left(1+\frac{\gamma-1}{2}\cdot Ma^2\right)^{\frac{\gamma}{\gamma-1}} \tag{S.33}$$

$$T_2 = T_s \cdot \left(1+\frac{\gamma-1}{2}\cdot Ma^2\right) \tag{S.34}$$

Supplementary Note 19. BC mass emission dataset

In the original AEIC model, the BC mass emissions were calculated based on the FOA3[24] or FOX[26] methods. In this study, we adopt the BC mass emission indices provided by the EEA emission inventory guidebook[40], which was compiled using the Advanced Emission Model (AEM) for nearly 300 different types of aircrafts during LTO (Landing and Take-Off) and CCD (Climb/Cruise/Descent) phases at various flight distances. The dataset contains the fuel consumptions and the gaseous emission indices. In the AEM model, the BC emissions are also estimated by the SN dependent FOA3 method[24], but the missing SN data in the ICAO Engine Emissions Databank [19] have already been estimated based on the algorithm developed by German Aerospace Center (DLR) and the cruise emissions are also corrected for the atmospheric environment at high altitudes[66].

The EEA data provides the total emissions respectively for the whole LTO and CCD phases. The flight conditions (e.g. fuel flow rate, speed and altitude) are not constant during LTO or CCD. The different flight conditions influence the BC emission and mass to number conversion. In order to better estimate the number emissions, the EEA mass emission data for LTO and CCD phases are further divided into seven sub-phases, taxi/take-off/climb-out/approach for the LTO cycle and climb/cruise/descent for the CCD phase.

In order to separate the data, we first estimate the durations of the sub-phases at various flight distances based on Automatic Dependent Surveillance-Broadcast (ADS-B) data (Supplementary Note 20). Then the typical speed of each sub-phase is also approximated based on the ADS-B data (Supplementary Note 21). The fuel flow and thrust setting are analyzed based on the Flight Data

Recorder (FDR) data (Supplementary Note 22). At last, the separation method is introduced (Supplementary Note 23).

Supplementary Note 20. Flight duration

For the LTO cycle, the duration and fuel flow rates follow the ICAO default settings. The case of the CCD phase is more complicated due to the lack of default setting. In order to analyze the flight durations of each sub-phase during CCD, more than 13000 records of the Automatic Dependent Surveillance-Broadcast (ADS-B) are first collected from the OpenSky network (https://opensky-network.org/). The data contain about 1500 regular flight routes on 9 different days (operated by American Airlines and Lufthansa), which are shown in Supplementary Figure 21.

The flight routes are classified into three groups according to the duration of CCD: short (<60 min), medium (60 to 120 min) and long (>120 min), because the shares of the sub-phases significantly change with different flight durations. The cruise duration is extracted for all flights by an algorithm based on the flight vertical velocity and altitude. In each group, a robust linear regression is utilized to estimate the cruise duration as shown in Supplementary Figure 25. The absolute value of the intercept can be seen as the total duration of climb and descend. The durations of the climb and descent are determined based on the statistical ratio between them. The duration ratios between descent and climb phases are estimated to be 1.51, 1.32 and 1.26 respectively for short, medium and long haul flights.

Supplementary Note 21. Flight speed

For different flight routes (short, medium and long), the typical speeds (mean speeds) of the sub-phases in CCD are first respectively estimated based on the ADS-B data, as shown in Supplementary Figure 23. When estimating the number emission for a specific aircraft-route pair, the typical speeds (V_C^0, V_{CR}^0, V_D^0 with subscripts 'C' for Climb, 'CR' for Cruise and 'D' for Descent) are

modified by a correction factor (f_V) for each flight to make the calculated flight distance equal to the route length (D_{Route}) between the departure and the arrival airports.

$$f_V = \frac{D_{Route}}{V_C^0 \cdot T_C + V_{CR}^0 \cdot T_{CR} + V_D^0 \cdot T_D} \tag{S.35}$$

$$(V_C^{flight}, V_{CR}^{flight}, V_D^{flight}) = f_V \cdot (V_C^0, V_{CR}^0, V_D^0) \tag{S.36}$$

The cruise altitudes are provided in the EEA dataset. During the climb and descent phase, the mean value between 3000 ft and the cruise altitude is utilized as the reference flight high for the calculation.

It is difficult to distinguish the sub-phases of LTO in the ADS-B data, so the typical flight speeds of the sub-phases in the LTO cycle (Supplementary Figure 19) are statistically analyzed based on 901 records of Flight Data Recorder (FDR)[67] (Supplementary Figure 20), which provides the identifications for each fight phases. The mean values are utilized.

Supplementary Note 22. Fuel flow rate and thrust setting

The thrust setting during LTO cycles follows the ICAO default settings. The typical fuel flow rates during CCD are analyzed using the FDR dataset[67]. One example of the fuel flows normalized by the maximum flow rate (taking-off) during CCD is shown in Supplementary Figure 20. The typical normalized fuel flow rates of different sub-phases (FF^0) are estimated based on all the FDR data (Supplementary Figure 24). Similar as the estimation of flight speed, the typical fuel consumptions are also modified by a correction factor (f_{FF}) for each specific flight to make the total fuel consumption (F_{CCD}) during CCD phases the same as that defined in the EEA dataset.

$$f_{FF} = \frac{F_{CCD}}{FF_C^0 \cdot T_C + FF_{CR}^0 \cdot T_{CR} + FF_D^0 \cdot T_D} \tag{S.37}$$

$$(FF_C^{flight}, FF_{CR}^{flight}, FF_D^{flight}) = f_{FF} \cdot (FF_C^0, FF_{CR}^0, FF_D^0) \tag{S.38}$$

Supplementary Note 23. Division into sub-phases

Based on the above data, the BC mass emissions are estimated following the basic methods in the AEM model[66] for each sub-phase. The calculated results are not exactly the same as those in the EEA dataset due to the different input data and the missing of detailed correction method for the emissions at cruise altitude. As a result, the BC mass emissions in the EEA dataset are divided into the seven sub-phases only based on the shares of them, which are obtained by our calculations. In this way, the total amounts of BC mass emissions during LTO and CCD phases remain the same as those in the original EEA data.

Supplementary Note 24. Summary of the required input data

All the required input data are shown in Supplementary Table 11. The mass emission indices of BC are available from Annex 5-Master emission calculator of the EMEP/EEA air pollutant emission inventory guidebook 2016[40]. The flight altitude, speed and fuel flow rate of each sub-phase are estimated based on the EEA data, FDR and ADS-B data. The ambient temperature, pressure and sound speed at the flight altitude are estimated based on the International Standard Atmosphere (ISA)[69], which are required for the calculation of T_3. The engine data is from the ICAO Engine Emissions Databank[19].

Supplementary References

70 Petzold, A., Döpelheuer, A., Brock, C. A. & Schröder, F. In situ observations and model calculations of black carbon emission by aircraft at cruise altitude. Journal of Geophysical Research: Atmospheres 104, 22171-22181, doi:10.1029/1999jd900460 (1999).

71 Sorensen, C. M. The Mobility of Fractal Aggregates: A Review. Aerosol Science and Technology 45, 765-779, doi:10.1080/02786826.2011.560909 (2011).

72 Heintzenberg, J. Properties of the Log-Normal Particle Size Distribution. Aerosol Science and Technology 21, 46-48, doi:10.1080/02786829408959695 (1994).
