# Supplementary material for: A number-based inventory of size-resolved black carbon particle emissions by global civil aviation
Source: Nat Commun. 2019 Feb 1;10:534. doi: 10.1038/s41467-019-08491-9 (PMC6358618; doi:10.1038/s41467-019-08491-9)
Supplement: Supplementary file 3 — Description of Additional Supplementary Files [file 41467_2019_8491_MOESM3_ESM.docx]

**Descriptions for the Supplementary Data**

File: Supplementary Data 1

Format: MATLAB ‘mat’ binary data format.

| No. | Variable Name | Dimension | Unit | Description |
| --- | --- | --- | --- | --- |
| 1 | num_bin_grid_ensemble_mean | 16×180×360  (L×M×N) | # | The mean of the 100 Monte Carlo calculations for the black carbo (BC) particle number emission (#) in 16 different size bins from 10nm to 6309nm as shown in Supplementary Table7 in each grid box (1 degree latitude×1 degree longitude):   - 1^st^ dimension (L=16): 16 size bins as shown in Supplementary Table7; - 2^nd^ dimension (M=180): latitude coordinates from −90° to 90° with a resolution of 1 degree; - 3^rd^ dimension (N=360): longitude coordinates from −180° to 180° with a resolution of 1 degree. |
| 2 | num_grid_ensemble_mean | 180×360  (M×N) | # | The mean of the 100 Monte Carlo calculations for the aviation BC particle number emissions in each grid box (1 degree latitude×1 degree longitude):   - 1^st^ dimension (M=180): latitude coordinates from −90° to 90° with a resolution of 1 degree; - 2^nd^ dimension (N=360): longitude coordinates from −180° to 180° with a resolution of 1 degree. |
| 3 | mass_grid | 180×360  (M×N) | kg | The aviation BC mass emissions in each grid box (1 degree latitude×1 degree longitude):   - 1^st^ dimension (M=180): latitude coordinates from −90° to 90° with a resolution of 1 degree; - 2^nd^ dimension (N=360): longitude coordinates from −180° to 180° with a resolution of 1 degree. |
| 4 | fuel_grid | 180×360  (M×N) | kg | The aviation fuel consumption in each grid box (1 degree latitude×1 degree longitude):   - 1^st^ dimension (M=180): latitude coordinates from −90° to 90° with a resolution of 1 degree; - 2^nd^ dimension (N=360): longitude coordinates from −180° to 180° with a resolution of 1 degree. |
| 5 | gmd_all_ensemble | 100 | nm | The fitted GMDs (nm) of the total BC particles from the global aviation emissions for the 100 Monte Carlo calculations. |
| 6 | gmd_ccd_sub_ensemble | 3×100 | nm | The fitted GMDs (nm) of the BC particles from the global aviation emissions during the three CCD sub-phases (climb, cruise and descent) for the 100 Monte Carlo calculations.   - 1^st^ dimension (K=3): three CCD sub-phases, (1) climb, (2) cruise and (3) descent; - 2^nd^ dimension (M=100): the 100 Monte Carlo calculations. |
| 7 | gmd_ccd_all_ensemble | 100 | nm | The fitted GMDs (nm) of the BC particles from the global aviation emissions during the whole CCD phase for the 100 Monte Carlo calculations. |
| 8 | gmd_lto_sub_ensemble | 4×100 | nm | The fitted GMDs (nm) of the BC particles from the global aviation emissions during the four LTO sub-phases (taxi, take-off, climb-out and approach) for the 100 Monte Carlo calculations.   - 1^st^ dimension (K=4): four LTO sub-phases, (1) taxi, (2) take-off, (3) climb-out and (4) approach; - 2^nd^ dimension (M=100): the 100 Monte Carlo calculations. |
| 9 | gmd_lto_all_ensemble | 100 | nm | The fitted GMDs (nm) of the BC particles from the global aviation emissions during the whole LTO phase for the 100 Monte Carlo calculations. |
| 10 | gsd_all_ensemble | 100 | / | The fitted GSDs of the total BC particles from the global aviation emissions for the 100 Monte Carlo calculations. |
| 11 | gsd_ccd_sub_ensemble | 3×100 | / | The fitted GSDs of the BC particles from the global aviation emissions during the three CCD sub-phases (climb, cruise and descent) for the 100 Monte Carlo calculations.   - 1^st^ dimension (K=3): three CCD sub-phases, (1) climb, (2) cruise and (3) descent; - 2^nd^ dimension (M=100): the 100 Monte Carlo calculations. |
| 12 | gsd_ccd_all_ensemble | 100 | / | The fitted GSDs of the BC particles from the global aviation emissions during the whole CCD phase for the 100 Monte Carlo calculations. |
| 13 | gsd_lto_sub_ensemble | 4×100 | / | The fitted GSDs of the BC particles from the global aviation emissions during the four LTO sub-phases (taxi, take-off, climb-out and approach) for the 100 Monte Carlo calculations.   - 1^st^ dimension (K=4): four LTO sub-phases, (1) taxi, (2) take-off, (3) climb-out and (4) approach; - 2^nd^ dimension (M=100): the 100 Monte Carlo calculations. |
| 14 | gsd_lto_all_ensemble | 100 | / | The fitted GSDs of the BC particles from the global aviation emissions during the whole LTO phase for the 100 Monte Carlo calculations. |
| 15 | num_ccd_sub_ensemble | 3×100 | # | The number of the BC particles from the global aviation emissions during the three CCD sub-phases (climb, cruise and descent) for the 100 Monte Carlo calculations.   - 1^st^ dimension (K=3): three CCD sub-phases, (1) climb, (2) cruise and (3) descent; - 2^nd^ dimension (M=100): the 100 Monte Carlo calculations. |
| 16 | num_lto_sub_ensemble | 4×100 | # | The number of the BC particles from the global aviation emissions during the four LTO sub-phases (taxi, take-off, climb-out and approach) for the 100 Monte Carlo calculations:   - 1^st^ dimension (K=4): four LTO sub-phases, (1) taxi, (2) take-off, (3) climb-out and (4) approach; - 2^nd^ dimension (M=100): the 100 Monte Carlo calculations. |
| 17 | mass_ccd_sub | 3 | kg | The aviation BC mass emissions during the three CCD sub-phases: (1) climb, (2) cruise and (3) descent. |
| 18 | mass_lto_sub | 4 | kg | The aviation BC mass emissions during the three LTO sub-phases: (1) taxi, (2) take-off, (3) climb-out and (4) approach. |
| 19 | fuel_ccd_sub | 3 | kg | The aviation fuel consumption during the three CCD sub-phases: (1) climb, (2) cruise and (3) descent. |
| 20 | fuel_lto_sub | 4 | kg | The aviation fuel consumption during the three LTO sub-phases: (1) taxi, (2) take-off, (3) climb-out and (4) approach. |
| 21 | num_bin_ccd_sub_ensemble | 16×3×100  (L×M×N) | # | The number of the BC particles in 16 different size bins from 10nm to 6309nm as shown in Supplementary Table 7 from the global aviation emissions during the three CCD sub-phases (climb, cruise and descent) for the 100 Monte Carlo calculations:   - 1^st^ dimension (L=16): 16 size bins as shown in Supplementary Table 7; - 2^nd^ dimension (M=3): three CCD sub-phases, (1) climb, (2) cruise and (3) descent; - 3^rd^ dimension (N=100): the 100 Monte Carlo calculations. |
| 22 | num_bin_lto_sub_ensemble | 16×4×100  (L×M×N) | # | The number of the BC particles in 16 different size bins from 10nm to 6309nm as shown in Supplementary Table 7 from the global aviation emissions during the three LTO sub-phases (taxi, take-off, climb-out and approach) for the 100 Monte Carlo calculations:   - 1^st^ dimension (L=16): 16 size bins as shown in Supplementary Table 7; - 2^nd^ dimension (M=4): four LTO sub-phases, (1) taxi, (2) take-off, (3) climb-out and (4) approach; - 3^rd^ dimension (N=100): the 100 Monte Carlo calculations. |
